# Prevalence of soil-transmitted helminth infections in HIV patients: a systematic review and meta-analysis

**DOI:** 10.1038/s41598-023-38030-y

**Published:** 2023-07-08

**Authors:** Kumari Akanksha, Ashu Kumari, Omprokash Dutta, Ajay Prasanth, Farah Deeba, Nasir Salam

**Affiliations:** 1 Department of Microbiology, Central University of Punjab, Bathinda, Punjab 151401 India; 2 Centre for Interdisciplinary Research in Basic Sciences, Jamia Millia Islamia, New Delhi, 110025 India; 3 Department of Biosciences, Jamia Millia Islamia, New Delhi, 110025 India

**Keywords:** Infectious diseases, Epidemiology

## Abstract

Soil-transmitted Helminth (STH) infections have been found associated with people living with human immunodeficiency virus (HIV) but little is known about the overall burden of STH coinfection in HIV patients. We aimed to assess the burden of STH infections among HIV patients. Relevant databases were systematically searched for studies reporting the prevalence of soil-transmitted helminthic pathogens in HIV patients. Pooled estimates of each helminthic infection were calculated. The odds ratio was also determined as a measure of the association between STH infection and the HIV status of the patients. Sixty-one studies were finally included in the meta-analysis, consisting of 16,203 human subjects from all over the world. The prevalence of *Ascaris lumbricoides* infection in HIV patients was found to be 8% (95% CI 0.06, 0.09), the prevalence of *Trichuris trichiura* infection in HIV patients was found to be 5% (95% CI 0.04, 0.06), the prevalence of hookworm infection in HIV patients was found to be 5% (95% CI 0.04, 0.06), and prevalence of *Strongyloides stercoralis* infection in HIV patients was found to be 5% (95% CI 0.04, 0.05). Countries from Sub-Saharan Africa, Latin America & Caribbean and Asia were identified with the highest burden of STH-HIV coinfection. Our analysis indicated that people living with HIV have a higher chance of developing *Strongyloides stercoralis* infections and decreased odds of developing hookworm infections. Our findings suggest a moderate level of prevalence of STH infections among people living with HIV. The endemicity of STH infections and HIV status both are partially responsible for the burden of STH-HIV coinfections.

## Introduction

Soil-transmitted helminth infections affect nearly 1.5 billion people living in low- and middle-income countries^1^. These infections are caused by four different pathogens, *Ascaris lumbricoides*, *Trichuris trichiura*, Hookworm, and *Strongyloides stercoralis*^2^. Infection with STH is often associated with diarrhoea, anaemia, malnutrition, and stunted growth among children causing substantial morbidity and mortality^3,4^. *A. lumbricoides* infection occurs by ingestion of embryonated eggs through contaminated, food, water, and soil. The larvae migrate via the liver and lung, ultimately establishing in the small intestine. The female produces eggs that are passed into the faeces. Symptoms like abdominal stress, diarrhoea, weight loss, and weakness appear only in individuals with a heavy parasitic load. Cognitive impairment in children is often associated with *Ascaris* infection in endemic areas^5,6^. The ingested eggs of *T. trichiura* hatch in the small intestine releasing the larvae which establish as adult worms in the colon. Most individuals with low parasite burden are asymptomatic but those with heavy parasite burden, particularly children can show symptoms of abdominal discomfort, diarrhoea, and rectal prolapse^7^. Hookworm takes residence in the small intestine attaching itself to the intestinal wall and feeding on the host blood. Blood loss at the attachment site results in iron deficiency anaemia and low haemoglobin levels^8^. The larvae of *S. stercoralis* enters via skin penetration and individuals migrate through body tissues, eventually settling in the small intestine where adult female produces eggs. These eggs hatch in the last part of the bowel and they are excreted with faeces. Some of these larvae can autoinfect the host via penetration of perianal skin causing long-term infection of the host. Chronic infections with *S. stercoralis* are associated with gastrointestinal symptoms—diarrhoea, abdominal pain, and distension, respiratory symptoms—asthma and dyspnoea, skin associated symptoms—itching and urticaria^9,10^. Socioeconomic status, sanitation, and personal hygiene are common risk factors responsible for the acquisition of STH infections^11^. The geographical regions of the world, most affected by STHs, are also affected by HIV infections. Currently, 38.4 million people are affected by HIV globally and 650,000 people died from HIV-related causes in 2021^12^. Infection with HIV results in weakened immunity leading to the acquisition of several opportunistic and non-opportunistic pathogens exacerbating the illness^13,14^. Diarrhoea among HIV patients is often the result of infection with STH pathogens.

Interaction between STHs and their human host might influence susceptibility toward other pathogens. Many reports have suggested that helminth infection contributes to compromised protective immunity against *Plasmodium* and *Mycobacterium*^15^. Despite the high prevalence of STH and HIV infections, the effect of STH infections on HIV acquisition and transmission, disease progression, and management remain inconclusive. It is possible that STH infection contributes to increased susceptibility to HIV infection either directly through an immunological mechanism or indirectly via causing malnutrition^16^. It has been observed that coinfection with STHs among people living with HIV contributes to exacerbating HIV infection by impairing T_H_1 immunity which is necessary for clearing the viral load^17–19^. Studies on the effects of deworming, however, present conflicting results. In some reports, it has been observed that deworming did not affect the severity of HIV infection^20,21^. Others show a reduction in viral load after deworming and a delay in disease progression^22–24^. Infection with both pathogens simultaneously affects the general health and well-being of the patient and timely interventions among the affected population are required for the control of infection. STH infections could be a potential risk factor for HIV acquisition and disease progression and deworming may be a helpful intervention. With a continued focus on the control of HIV and renewed efforts towards the elimination of NTDs as outlined in Sustainable Development Goals, it is crucial to understand the co-prevalence and geographical distribution of STHs and HIV. The goal of the present study is to analyse the global burden of STH infections among HIV patients by systematically reviewing and analysing the literature available on the topic.

## Methods

### Search strategy

The systematic review was carried out based on PRISMA (Preferred Reporting Items for Systematic Reviews and Meta-analysis) guidelines. Two authors, KA and NS searched PubMed and Web of Science without including a time frame in the literature search up to October 2021 to retrieve studies reporting the prevalence of HIV and any of the STH. The following keywords were used: “HIV” or “AIDS” AND “Helminth” or “*Ascaris*” or “Whipworm” or “Hookworm” or “*Trichuris*” or “*Necator*” or “*Ancylostoma*” or “*Strongyloides*” or “Threadworm.” All the studies were transferred to the Zotero citation manager. Initially, the duplicates were removed by the inbuilt feature of Zotero and later by manually screening the remaining studies.

### Selection criteria

We searched without any bar on the nature and geographical origin of the studies. Preliminary screening was done based on a review of the title and abstract of the studies. The full text of potentially relevant studies was further screened for data on the prevalence of HIV and STH coinfection. Studies reporting the number or percentage of STHs among HIV patients were considered eligible for data extraction.

### Study selection

The quality of the studies included in the final meta-analysis was assessed using Joanna Briggs Institute (JBI) criteria. The meta-analysis included studies with a rating of 7 or higher. KA carried out the quality assessment which was further verified by NS and FD. All the authors discussed and arrived at a consensus in case of any disagreements.

### Data extraction

The data was carefully extracted from the eligible studies and verified by multiple authors to avoid any discrepancies. KA and NS extracted the data while AK, OP, AP, and FD independently verified the extracted data The extracted data included the following information: author, year, study design, location, sample size, helminth prevalence (number or percentage), and diagnostic test for both HIV and the STH pathogens identified.

### Statistical analysis

Data analysis was done using Review Manager (RevMan) Version 5.4. The Cochrane Collaboration, 2020. For the determination of pooled prevalence from multiple studies, the random effects model was employed and it was calculated with a 95% confidence interval. Heterogeneity among studies was calculated using Cochran’s Q test and I^2^ statistics, with I^2^ > 50% considered as significant heterogeneity^25^. Subgroup analyses were conducted to explore the source of heterogeneity, stratified by geographical location. Funnel plots were generated to evaluate publication bias.

## Results

**Figure 1:**
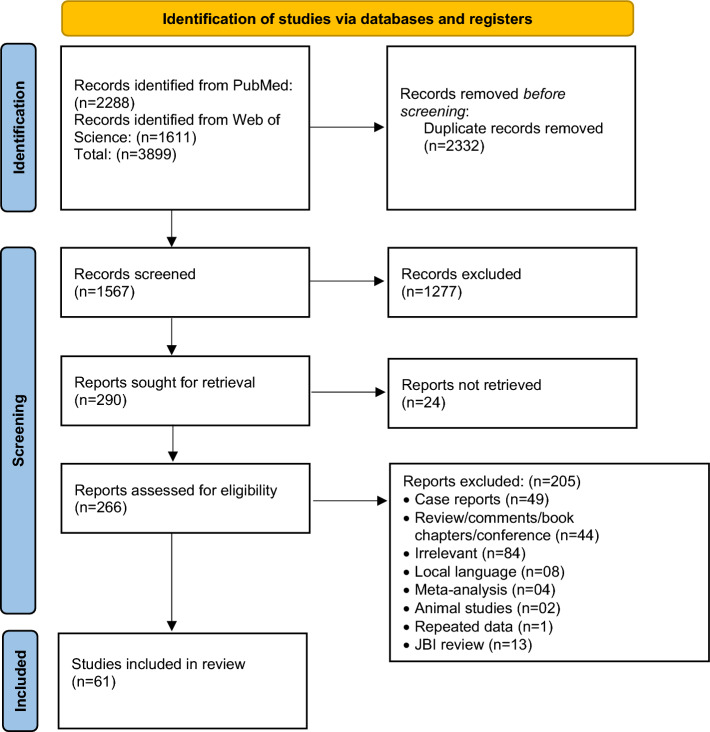
PRISMA protocol for the study selection process.

Initial searches yielded 1567 unique studies out of which the full text of 266 studies was assessed for eligibility. The study selection process was based on PRISMA guidelines (Fig. 1, Supp. Fig. 1). After excluding irrelevant studies and applying the JBI checklist, 61 studies were included in the final systematic review and meta-analysis (Supp. Table 1)^26–86^. Eleven of the included papers were rated 9 stars i.e., they fulfilled all the criteria mentioned in the JBI checklist, 31 papers were rated 8, and 19 papers were rated 7. Thirteen papers were excluded from the study as they were rated < 7 (supp. Tables 2–3). Thirteen countries from Sub-Saharan Africa (Nigeria, Ethiopia, Mozambique, South Africa, Malawi, Tanzania, Guinea-Bissau, Guinea, Kenya, Zambia, Uganda, Cameroon, and the Republic of Congo) Three countries from Latin America & Caribbean region (Cuba, Brazil, and Venezuela), seven countries from Asia (Laos, Thailand, Malaysia, India, China, Nepal, and Iran) two countries from Europe and North America (Ireland and the United States of America) reported the prevalence of coinfection with STHs in HIV infected patients (Supp. Table 3). Twenty-four studies reported all four parasites coinfecting HIV-positive patients, 17 studies reported at least three, 9 studies reported at least two, and 11 studies reported a single parasite coinfection associated with HIV-positive patients. The most common method for the diagnosis of HIV infection was ELISA, while all except one study used microscopy as the diagnostic method for parasite detection. Out of 61 studies, fifty-six were carried out after the year 2000 and the earliest reported study was from 1992.

**Figure 2:**
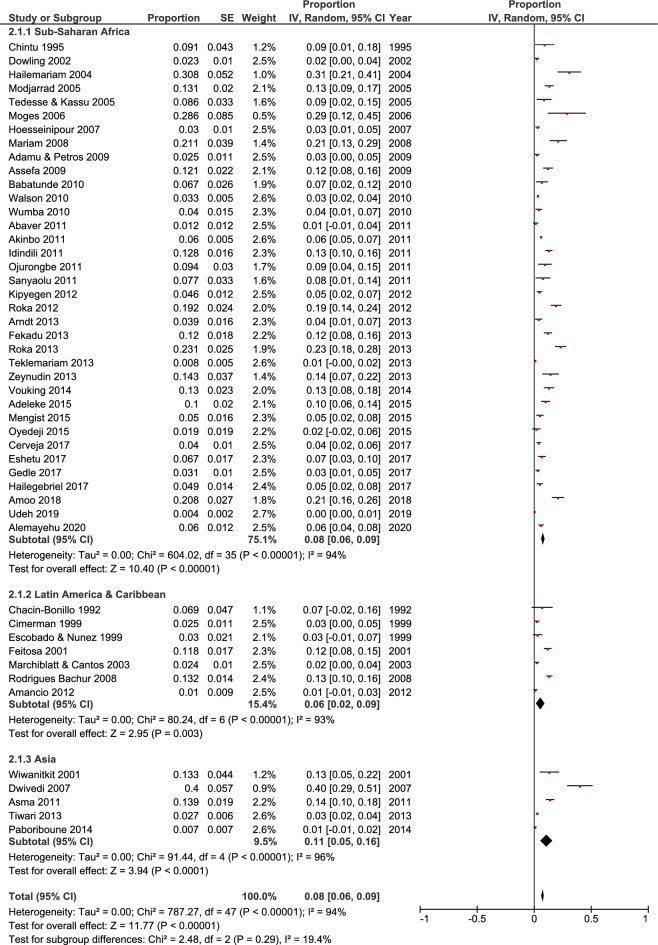
Random effects analysis for pooled prevalence estimate of *A. lumbricoides* (AL) infection in HIV patients.

**Figure 3:**
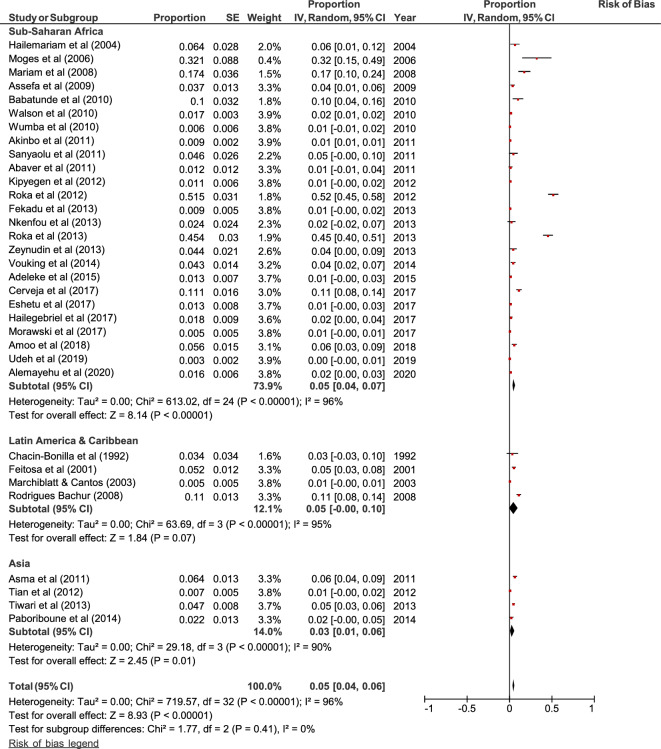
Random Effects analysis for pooled prevalence estimate of *T. trichiura* (TT) infection in HIV patients.

**Figure 4:**
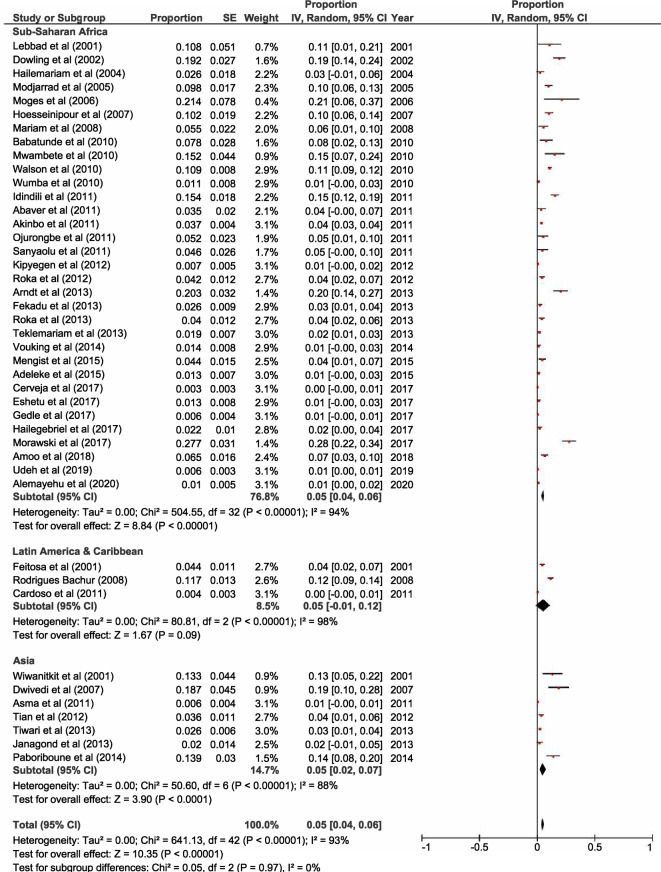
Random Effects analysis for pooled prevalence estimate of Hookworm infection in HIV patients.

**Figure 5:**
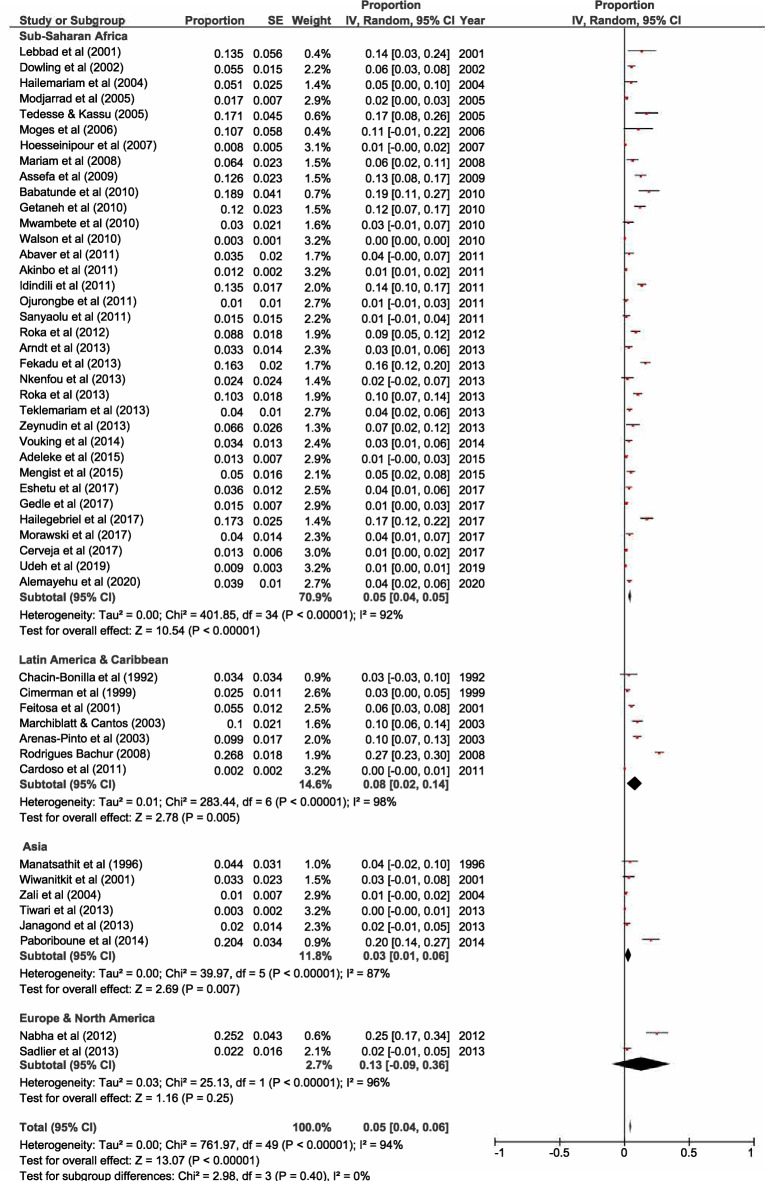
Random Effects analysis for pooled prevalence estimate of *S. stercoralis* (SS) infection in HIV patients.

Forty-eight out of 61 selected studies showed the prevalence of HIV/*A. lumbricoides* coinfection. The estimated pooled prevalence of *A. lumbricoides* infection was found to be 8% (95% CI 0.06, 0.09) overall, 8% (95% CI 0.06, 0.09) in Sub-Saharan Africa, 6% (95% CI 0.02, 0.09) in Latin America & Caribbean and 11% (95% CI 0.05, 0.16) in Asia (Fig. 2). Thirty-three out of 61 selected studies showed the prevalence of HIV/*T. trichiura* coinfection. The estimated pooled prevalence was found to be 5% (95% CI 0.04, 0.06) overall, 5% (95% CI 0.04, 0.07) in Sub-Saharan Africa, 5% (95% CI 0, 0.1) in Latin America & Caribbean and 3% (95% CI 0.01, 0.06) in Asia (Fig. 3). Forty-three out of 61 studies showed the prevalence of HIV/ Hookworm coinfection. The estimated pooled prevalence was found to be 5% (95% CI 0.04, 0.06) overall, 5% (95% CI 0.04, 0.06) in Sub-Saharan Africa, 5% (95% CI − 0.01, 0.12) in Latin America & Caribbean and 5% (95% CI 0.02, 0.07) in Asia (Fig. 4). Fifty out of 61 studies showed the prevalence of HIV/*S. stercoralis* coinfection. The estimated pooled prevalence was found to be 5% (95%CI 0.04, 0.05) overall, 5% (95% CI 0.04, 0.05) in Sub-Saharan Africa, 8% (95% CI 0.02, 0.14) in Latin America & Caribbean, 3% (95% CI 0.01, 0.06) in Asia and 13% (95% CI − 0.09, 0.36) in Europe and North America (Fig. 5). Tanzania, Guinea-Bissau, Guinea, and Uganda from Sub-Saharan Africa, India from Asia, and an immigrant sample group in the USA from North America are the only populations that showed a prevalence of 10% or higher for STH infections in people living with HIV (supp. Figs. 2–26, Supp. Table 2). In general, the number of studies reported from Sub-Saharan Africa was more in numbers as compared to Latin America & Caribbean, Asia and Europe, and North America. We did not find any studies from the Middle East and North African region. There was an implication of publication bias as indicated by asymmetrical funnel plots (supp. Figs. 27–30).

**Figure 6:**
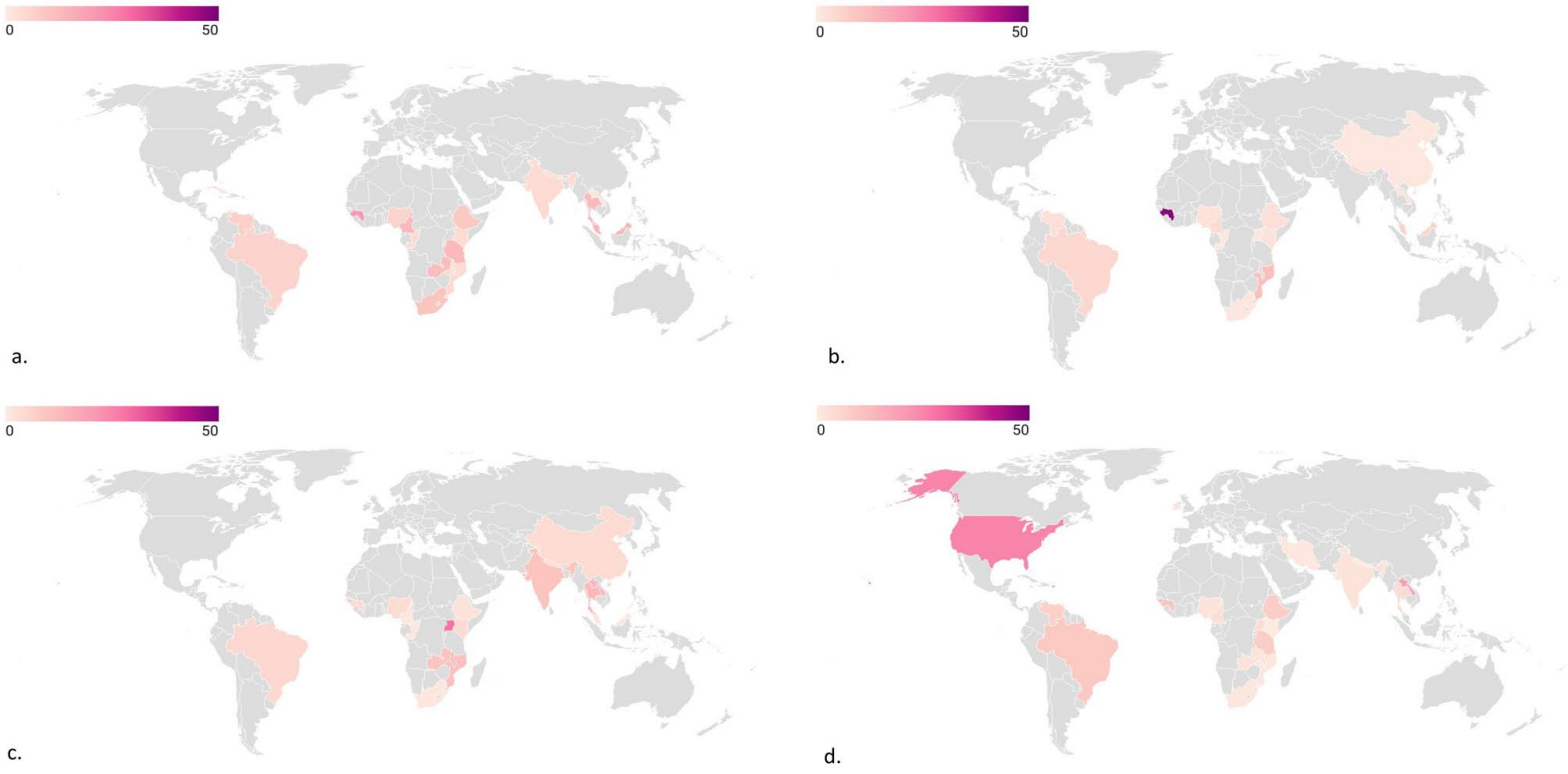
Map of STH infections in HIV-infected people worldwide a. *A. lumbricoides*/HIV coinfections b. *T. trichiura*/HIV coinfections c. Hookworm/HIV coinfections d. *S. stercoralis*/HIV coinfections. Pooled prevalence and 95% CI are shown for each country.

We calculated the odds ratio to determine the correlation between HIV infection status and STH infection. The odds did not differ significantly for HIV-positive patients and either *A. lumbricoides* [OR 0.89 (95% CI 0.73, 1.09)] or *T. trichuris* coinfections [OR 0.96 (95% CI 0.65, 1.43)] (Supp. Figs. 31–32). HIV infections were associated with decreased odds of hookworm coinfections [OR 0.59 (95% CI 0.38, 0.92)] while HIV infections were associated with more than two-fold increased odds of developing *S. stercoralis* infections [OR 2.71 (95% CI 1.47, 5.00)] (Supp. Figs. 33–34). A map showing the prevalence of STH infections among HIV patients indicated 0–48% prevalence across 26 countries (Fig. 6).

## Discussion

People living with HIV are at a higher risk of developing enteric infections either due to shared epidemiology or dysregulated immunity. These infections hurt the condition of HIV patients contributing to HIV-associated morbidity. Many individual studies have been carried out reporting the prevalence of STH infections among people living with HIV, yet a comprehensive overview of their prevalence has not been analysed. This is the first systematic review to assess the prevalence of the four most important STHs among people living with HIV. After applying the JBI criteria, only 61 studies became eligible for our review. Our analysis indicated a moderate level of prevalence of the four STH pathogens among people living with HIV. Microscopy of stool samples was uniformly used, except in two studies, for the identification of STH infections indicating current infections. *A. lumbricoides* which is the predominant helminthic infection globally showed the highest prevalence among HIV patients, with Sub-Saharan Africa and Asia showing maximum prevalence which is similar to other reports where the global prevalence was estimated to be 11.01%^5^. The prevalence among endemic regions as assessed in past studies and our data indicate that HIV infection does not necessarily makes patients more vulnerable to roundworm infection. It is rather associated with the endemicity than the immune status of the patient. Similarly, *T. trichiura* prevalence also confirms earlier findings with a 15.3% prevalence reported in Asia alone^7^. Apart from *A. lumbricoides* all the other three STH indicated an overall prevalence of 5% with a modest difference across population groups. Two studies conducted on immigrants from STH-endemic countries showed a pooled prevalence of 13% of *S. stercoralis* infections among HIV patients. As HIV infection is associated with impaired immunity which contributes to the development of several opportunistic infections, we determined the odds ratio of developing STH infections among HIV patients. Our analysis indicated that there was no significant association between HIV status and *A. lumbricoides* and *T. trichiura* coinfection indicating that infection with both these pathogens is independent of the HIV status of the patients and most likely the result of shared epidemiology. However, we did find a significant association between HIV status and hookworm infection indicating people living with HIV have a decreased risk of developing hookworm infection. These results are consistent with previously published reports where HIV infection was associated with lower hookworm infection rates^20,87^. This inverse relation between HIV and hookworm in previous studies and our results can be explained by HIV-induced enteropathy that damages the gastrointestinal tract and possibly creates an inhospitable environment for hookworm survival^88,89^. It was only in the case of *S. stercoralis* infection that we observed increased odds associated with HIV status. People living with HIV are more than twice likely to develop *S. stercoralis* infection which is in line with previously published reports about the prevalence and severity of this pathogen in immunosuppressed patients^90,91^. However, HIV-induced immunosuppression does not lead to disseminated disease or hyperinfection in the case of *S. stercoralis* infection possibly due to an increase in Th2 cytokines and indirect larval development that reduces the possibility of autoinfection.^92,93^ Majority of the studies considered in the final analysis were carried out on diverse age groups and both genders consequently gender or age subgroup analysis was not carried out. A few studies reported more than 50% prevalence of STH infection among HIV patients. Both studies by Roka et al. from Guinea, Dwivedi et al. from India, and Mariam et al. from Ethiopia, indicated a preponderance of environmental factors, geographical distribution, and behavioral patterns rather than HIV status as the reason for high prevalence.

Our meta-analysis has several limitations. The review protocol was not registered in International prospective register of systematic reviews (PROSPERO) as it was overloaded with pandemic related systematic reviews at the time and it would have delayed the registration and review of our protocol^94,95^. Several potentially relevant studies were identified through a systematic search of the databases but the full text of many of them was not available increasing the possibility of missing out on important data sets. Included data were from 26 countries and mostly concentrated in Sub-Saharan Africa, Latin America & Caribbean, and Asia. Two of the studies reported from Europe and North America tested immigrant populations without any information about their countries of origin and one of them found a very high prevalence of *S. stercoralis* infection underlining the need for increased attention on such population groups. Many countries and regions remain underrepresented emphasising the need for more robust surveillance of HIV-associated STH infections among people living in these countries. There was not enough data to analyse the correlation of altered CD4+ T cell numbers with the severity of STH or HIV infections, which would have provided evidence for the influence of these infections on each other. Significant heterogeneity was observed which is expected in global prevalence studies across different periods and geographical locations^96,97^.

Despite the limitations of the present study, it establishes the prevalence of STH infection among HIV patients with a clear link between HIV status and hookworm and *S. stercoralis* infection and makes the case for deworming as an intervention in STH endemic regions. Future studies are required to assess the long-term impact of STH and HIV coinfection on disease severity, progression, and prognosis.

## Conclusion

This is the first study that comprehensively analyses the prevalence of the four most prevalent STH infections among HIV patients showing a moderate level of prevalence. We found that HIV status is associated with increased chances of acquiring *S. stercoralis* infection and decreased chances of acquiring hookworm infections. People living with HIV in Sub-Saharan Africa, Latin America & Caribbean, Asia, and immigrant communities are at a higher risk of developing STH infections. Routine surveillance of HIV-infected people for STH infections should be carried out and provided therapeutic support for the treatment of these infections to improve the health and quality of their life.

## Supplementary Information

Supplementary Information.

## Data Availability

All data generated or analysed during this study are included in this published article [and its supplementary information files].
