# Supplementary material for: Prevalence of soil-transmitted helminth infections in HIV patients: a systematic review and meta-analysis
Source: Sci Rep. 2023 Jul 8;13:11055. doi: 10.1038/s41598-023-38030-y (PMC10329649; doi:10.1038/s41598-023-38030-y)
Supplement: Supplementary file 1 — Supplementary Information. [file 41598_2023_38030_MOESM1_ESM.pdf]

**Supplementary Data**

Contents------------------------------------------------------------------------------------ Page No.

Supp. Figure 1. Search results -----------------------------------------------------------------2
Supp. Table 1. Included studies table -----------------------------------------------------3-4
Supp. Figure 2-26. Country wise pooled prevalence------------------------------------5-29
Supp. Table 2. Combine table for country wise pooled prevalence-------------------30
Supp. Table 3. JBI score---------------------------------------------------------------------31-35
Supp. Figure 27. Funnel plot for pooled prevalence of AL in HIV patients-----------36
Supp. Figure 28. Funnel plot for pooled prevalence of TT in HIV patients-----------37
Supp. Figure 29. Funnel plot for pooled prevalence of HW in HIV patients---------38
Supp. Figure 30. Funnel plot for pooled prevalence of SS in HIV patients-----------39
Supp. Figure 31. Forest plot for odds of *A. lumbricoides* (AL) infection in HIV patients---------------------------------------------------------------------------------------------40
Supp. Figure 32. Forest plot for odds of *T. trichiura* (TT) infection in HIV patients---------------------------------------------------------------------------------------------41
Supp. Figure 33. Forest plot for odds of hookworm infection in HIV patients---------------------------------------------------------------------------------------------42
Supp. Figure 34. Forest plot for odds of S. *stercoralis* (SS) infection in HIV patients---------------------------------------------------------------------------------------------43

## PubMed Results

1. 16-10-2021- HIV, Helminth- 701
2. 16-10-2021- HIV, Ascaris-106
3. 16-10-2021- HIV, Whipworm- 73
4. 16-10-2021- HIV, Hookworm- 126
5. 16-10-2021- HIV, Trichuris- 63
6. 16-10-2021- HIV, Necator- 12
7. 16-10-2021- HIV, Ancylostoma- 14
8. 16-10-2021- HIV, Strongyloides- 265
9. 16-10-2021- HIV, Threadworm- 15
10. 16-10-2021- AIDS, Helminth- 468
11. 16-10-2021- AIDS, Ascaris- 67
12. 16-10-2021- AIDS, Whipworm- 49
13. 16-10-2021- AIDS, Hookworm- 74
14. 16-10-2021- AIDS, Trichuris- 42
15. 16-10-2021- AIDS, Necator- 11
16. 16-10-2021- AIDS, Ancylostoma- 08
17. 16-10-2021- AIDS, Strongyloides- 183
18. 16-10-2021- AIDS, Threadworm- 11

Total results- 2,288

## Web of Science results

11.10.2021

1. HIV + Helminth – 386
2. HIV + Ascaris – 96
3. HIV + Whipworm – 6
4. HIV + Hook worm – 110
5. HIV + Trichuris – 64
6. HIV + Necator – 14
7. HIV + Ancylostoma – 12
8. HIV + strongyloides – 184
9. HIV + threadworm – 1
10. AIDS + Helminth – 319
11. AIDS + Ascaris – 88
12. AIDS + Whipworm – 8
13. AIDS + Hook worm – 89
14. AIDS + Trichuris – 65
15. AIDS + Necator – 27
16. AIDS + Ancylostoma – 19
17. AIDS + strongyloides – 121
18. AIDS + threadworm – 2

Total results- 1,611

- Total results after removing duplication- 1,567

Supp. Figure 1.

| S. No. | Author | Country | Study design | HIV positive samples | Total STH infections | AL | TT | HW | SS | Diagnostic test for HIV | Parasite identification |
|---|---|---|---|---|---|---|---|---|---|---|---|
| Sub-Saharan Africa | | | | | | | | | | | |
| 1 | Abaver et al (2011)[26] | Nigeria | CS | 85 | 6 | 1 | 1 | 3 | 3 | RDT, WB | Microscopy |
| 2 | Akinbo et al (2011)[27] | Nigeria | CS | 2000 | 235 | 119 | 19 | 74 | 23 | ND | Microscopy |
| 3 | Amoo et al (2018)[28] | Nigeria | CS | 231 | 86 | 48 | 13 | 15 | 10 | RDT | Microscopy |
| 4 | Babatunde et al (2010)[29] | Nigeria | CS | 90 | 39 | 6 | 9 | 7 | 17 | ELISA | Microscopy |
| 5 | Ojurongbe et al (2011)[30] | Nigeria | CS | 96 | 15 | 9 | - | 5 | 1 | ND | Microscopy |
| 6 | Oyedeji et al (2015)[31] | Nigeria | PS | 52 | 1 | 1 | - | - | - | ELISA, WB | Microscopy |
| 7 | Sanyaolu et al (2011)[32] | Nigeria | CS | 65 | 12 | 5 | 3 | 3 | 1 | RDT | Microscopy |
| 8 | Udeh et al (2019)[33] | Nigeria | CCS | 891 | 20 | 4 | 3 | 5 | 8 | ND | Microscopy |
| 9 | Adamu and Petros (2009)[34] | Ethiopia | ND | 200 | 5 | 5 | - | - | - | ND | Microscopy |
| 10 | Alemayehu et al (2020)[35] | Ethiopia | CS | 383 | 55 | 23 | 6 | 4 | 15 | ND | Microscopy |
| 11 | Assefa et al (2009)[36] | Ethiopia | CS | 214 | 68 | 26 | 8 | 7 | 27 | ND | Microscopy |
| 12 | Eshetu et al (2017)[37] | Ethiopia | CS | 223 | 29 | 15 | 3 | 3 | 8 | ND | Microscopy |
| 13 | Fekadu et al (2013)[38] | Ethiopia | CS | 343 | 109 | 41 | 3 | 9 | 56 | ND | Microscopy |
| 14 | Gedle et al (2017)[39] | Ethiopia | CS | 323 | 17 | 10 | - | 2 | 5 | ND | Microscopy |
| 15 | Getaneh et al (2010)[40] | Ethiopia | CS | 192 | 23 | - | - | - | 23 | ND | Microscopy |
| 16 | Hailegebriel et al (2017)[41] | Ethiopia | CS | 226 | 59 | 11 | 4 | 5 | 39 | Serology | Microscopy |
| 17 | Hailemariam et al (2004)[42] | Ethiopia | CS | 78 | 35 | 24 | 5 | 2 | 4 | ELISA | Microscopy |
| 18 | Mengist et al (2015)[43] | Ethiopia | CS | 180 | 26 | 9 | - | 8 | 9 | ND | Microscopy |
| 19 | Moges et al (2006)[44] | Ethiopia | CS | 28 | 26 | 8 | 9 | 6 | 3 | ELISA | Microscopy, |
| 20 | Tadesse at al (2005)[45] | Ethiopia | CS | 70 | 18 | 6 | - | - | 12 | ELISA | Microscopy |
| 21 | Teklemariam et al (2013)[46] | Ethiopia | CS | 371 | 25 | 3 | - | 7 | 15 | ND | Microscopy |
| 22 | Mariam et al (2008)[47] | Ethiopia | CS | 109 | 55 | 23 | 19 | 6 | 7 | ELISA | Microscopy |
| 23 | Zeynudin et al (2013)[48] | Ethiopia | CS | 91 | 23 | 13 | 4 | - | 6 | ND | Microscopy |
| 24 | Cerveja et al (2017)[49] | Mozambique | CS | 371 | 62 | 15 | 41 | 1 | 5 | ND | Microscopy |
| 25 | Adeleke et al (2015)[50] | South Africa | CS | 231 | 32 | 23 | 3 | 3 | 3 | RT-PCR | Microscopy |
| 26 | Hosseinipour et al (2007)[51] | Malawi | ND | 266 | 37 | 8 | - | 27 | 2 | RDT, ELISA, RT-PCR | Microscopy |
| 27 | Dowling et al (2002)[52] | Malawi | CCS | 219 | 59 | 5 | - | 42 | 12 | ELISA | Microscopy |
| 28 | Idindili et al (2011)[53] | Tanzania | ND | 421 | 176 | 54 | - | 65 | 57 | ELISA | Microscopy |
| 29 | Mwambete et al (2010)[54] | Tanzania | CS | 66 | 12 | - | - | 10 | 2 | ND | Microscopy |
| 30 | Lebbad et al (2001)[55] | Guinea-Bissau | ND | 37 | 9 | - | - | 4 | 5 | ELISA | Microscopy |
| 31 | Roka et al (2012)[56] | Guinea | CS | 260 | 218 | 50 | 134 | 11 | 23 | ND | Microscopy |
| 32 | Roka et al (2013)[57] | Guinea | CS | 273 | 226 | 63 | 124 | 11 | 28 | ND | Microscopy |
| 33 | Arndt et al (2013)[58] | Kenya | CS | 153 | 42 | 6 | - | 31 | 5 | ND | Microscopy |
| 34 | Kipyegen et al (2012)[59] | Kenya | CS | 285 | 18 | 13 | 3 | 2 | - | ND | Microscopy |
| 35 | Walson et al (2010)[60] | Kenya | ND | 1541 | 249 | 51 | 26 | 168 | 4 | ND | Microscopy |
| 36 | Chintu et al (1995)[61] | Zambia | CS | 44 | 4 | 4 | - | - | - | ELISA | Microscopy |
| 37 | Modjarrad et al (2005)[62] | Zambia | PS | 297 | 73 | 39 | - | 29 | 5 | RDT | Microscopy |
| 38 | Morawski et al (2017)[63] | Uganda | CS | 202 | 65 | - | 1 | 56 | 8 | ND | PCR |

| | | | | | | | | | | | |
|---|---|---|---|---|---|---|---|---|---|---|---|
| 39 | Nkenfou et al (2013)[64] | Cameroon | CS | 42 | 002 | - | 1 | - | 1 | RDT, PCR | Microscopy |
| 40 | Vouking et al (2014)[65] | Cameroon | CS | 207 | 46 | 27 | 9 | 3 | 7 | ND | Microscopy |
| 41 | Wumba et al (2010)[66] | Republic of Congo | CS | 175 | 10 | 7 | 1 | 2 | - | ND | Microscopy |
| Latin America & Caribbean | | | | | | | | | | | |
| 42 | Escobado & Nunez (1999)[67] | Cuba | CS | 67 | 2 | 2 | - | - | - | ELISA, WB | Microscopy |
| 43 | Amancio et al (2012)[68] | Brazil | CS | 105 | 1 | 1 | - | - | - | WB | Microscopy |
| 44 | Cardoso et al (2011)[69] | Brazil | RS | 500 | 3 | - | - | 2 | 1 | ELISA, WB, NAH | Microscopy |
| 45 | Cimerman et al (1999)[70] | Brazil | ND | 200 | 10 | 5 | - | - | 5 | ELISA, WB | Microscopy |
| 46 | Feitosa et al (2001)[71] | Brazil | ND | 365 | 98 | 43 | 19 | 16 | 20 | WB | Microscopy |
| 47 | Marchi Blatt & Cantos (2003)[72] | Brazil | PS | 211 | 27 | 5 | 1 | - | 21 | ELISA, WB, IF | Microscopy |
| 48 | Rodrigues Bachur et al (2008)[73] | Brazil | RS | 582 | 365 | 77 | 64 | 68 | 156 | ND | Microscopy |
| 49 | Arenas-Pinto et al (2003)[74] | Venezuela | CS | 304 | 30 | - | - | - | 30 | ND | Microscopy, |
| 50 | Chacin-Bonilla et al (1992)[75] | Venezuela | ND | 29 | 4 | 2 | 1 | - | 1 | ND | Microscopic |
| Asia | | | | | | | | | | | |
| 51 | Paboriboune et al (2014)[76] | Laos | PS | 137 | 51 | 1 | 3 | 19 | 28 | ELISA, RDT | Microscopy |
| 52 | Manatsathit et al (1996)[77] | Thailand | PS | 45 | 2 | - | - | - | 2 | ND | Microscopy |
| 53 | Wiwanitkit et al (2001)[78] | Thailand | CS | 60 | 18 | 8 | - | 8 | 2 | ND | Microscopy |
| 54 | Asma et al (2011)[79] | Malaysia | ND | 346 | 72 | 48 | 22 | 2 | - | ND | Microscopy |
| 55 | Dwivedi et al (2007)[80] | India | CCS | 75 | 44 | 30 | - | 14 | - | ST | Microscopy |
| 56 | Janagond et al (2013)[81] | India | ND | 100 | 4 | - | - | 2 | 2 | ND | Microscopy |
| 57 | Tian et al (2012)[82] | China | ND | 302 | 13 | - | 2 | 11 | - | ELISA, WB | Microscopy |
| 58 | Tiwari et al (2013)[83] | Nepal | CS | 745 | 76 | 20 | 35 | 19 | 2 | ND | Microscopy |
| 59 | Zali et al (2004)[84] | Iran | CS | 206 | 2 | - | - | - | 2 | ELISA | Microscopy |
| Europe & North America | | | | | | | | | | | |
| 60 | Sadlier et al (2013)[85] | Ireland | PS | 90 | 2 | - | - | - | 2 | ND | ELISA |
| 61 | Nabha et al (2012)[86] | USA | PS | 103 | 26 | - | - | - | 26 | NAH | Microscopy |

Table 1. Included studies of STH infections in HIV positive patients.

CS-cross sectional, PS-prospective study, CCS-case control study, RDT-rapid diagnostic test (serology), ELISA-enzyme linked immunosorbent assay, WB-western blot, , RT-PCR-reverse transcriptase polymerase chain reaction, NAH-nucleic acid hybridisation, IF-Immunofluorescence, ST-serological test, ND-Not defined, AL-*A. lumbricoides*, TT-*T. trichiura*, HW-hookworm, SS-*S. stercoralis*

| Study or Subgroup | Proportion | SE | Weight | Proportion IV, Random, 95% CI | Year |
|---|---|---|---|---|---|
| **Pooled prevalence of AL** | | | | | |
| Babatunde et al (2010) | 0.067 | 0.026 | 1.7% | 0.07 [0.02, 0.12] | 2010 |
| Abaver et al (2011) | 0.012 | 0.012 | 4.3% | 0.01 [-0.01, 0.04] | 2011 |
| Akinbo et al (2011) | 0.06 | 0.005 | 6.3% | 0.06 [0.05, 0.07] | 2011 |
| Ojurongbe et al (2011) | 0.094 | 0.03 | 1.4% | 0.09 [0.04, 0.15] | 2011 |
| Sanyaolu et al (2011) | 0.077 | 0.033 | 1.2% | 0.08 [0.01, 0.14] | 2011 |
| Oyedeji et al (2015) | 0.019 | 0.019 | 2.7% | 0.02 [-0.02, 0.06] | 2015 |
| Amoo et al (2018) | 0.208 | 0.027 | 1.6% | 0.21 [0.16, 0.26] | 2018 |
| Udeh et al (2019) | 0.004 | 0.002 | 6.8% | 0.00 [0.00, 0.01] | 2019 |
| **Subtotal (95% CI)** | | | **26.0%** | **0.06 [0.03, 0.09]** | |
| Heterogeneity: Tau² = 0.00; Chi² = 176.18, df = 7 (P < 0.00001); I² = 96% | | | | | |
| Test for overall effect: Z = 3.68 (P = 0.0002) | | | | | |
| **Pooled prevalence of TT** | | | | | |
| Babatunde et al (2010) | 0.1 | 0.032 | 1.3% | 0.10 [0.04, 0.16] | 2010 |
| Abaver et al (2011) | 0.012 | 0.012 | 4.3% | 0.01 [-0.01, 0.04] | 2011 |
| Akinbo et al (2011) | 0.009 | 0.002 | 6.8% | 0.01 [0.01, 0.01] | 2011 |
| Sanyaolu et al (2011) | 0.046 | 0.026 | 1.7% | 0.05 [-0.00, 0.10] | 2011 |
| Amoo et al (2018) | 0.056 | 0.015 | 3.5% | 0.06 [0.03, 0.09] | 2018 |
| Udeh et al (2019) | 0.006 | 0.002 | 6.8% | 0.01 [0.00, 0.01] | 2019 |
| **Subtotal (95% CI)** | | | **24.4%** | **0.02 [0.01, 0.03]** | |
| Heterogeneity: Tau² = 0.00; Chi² = 21.97, df = 5 (P = 0.0005); I² = 77% | | | | | |
| Test for overall effect: Z = 3.14 (P = 0.002) | | | | | |
| **Pooled prevalence of HW** | | | | | |
| Babatunde et al (2010) | 0.078 | 0.028 | 1.6% | 0.08 [0.02, 0.13] | 2010 |
| Ojurongbe et al (2011) | 0.052 | 0.023 | 2.1% | 0.05 [0.01, 0.10] | 2011 |
| Sanyaolu et al (2011) | 0.046 | 0.026 | 1.7% | 0.05 [-0.00, 0.10] | 2011 |
| Abaver et al (2011) | 0.035 | 0.02 | 2.5% | 0.04 [-0.00, 0.07] | 2011 |
| Akinbo et al (2011) | 0.037 | 0.004 | 6.5% | 0.04 [0.03, 0.04] | 2011 |
| Amoo et al (2018) | 0.065 | 0.016 | 3.3% | 0.07 [0.03, 0.10] | 2018 |
| Udeh et al (2019) | 0.006 | 0.003 | 6.7% | 0.01 [0.00, 0.01] | 2019 |
| **Subtotal (95% CI)** | | | **24.4%** | **0.04 [0.02, 0.06]** | |
| Heterogeneity: Tau² = 0.00; Chi² = 55.48, df = 6 (P < 0.00001); I² = 89% | | | | | |
| Test for overall effect: Z = 3.77 (P = 0.0002) | | | | | |
| **Pooled prevalence of SS** | | | | | |
| Babatunde et al (2010) | 0.189 | 0.041 | 0.8% | 0.19 [0.11, 0.27] | 2010 |
| Abaver et al (2011) | 0.035 | 0.02 | 2.5% | 0.04 [-0.00, 0.07] | 2011 |
| Akinbo et al (2011) | 0.012 | 0.002 | 6.8% | 0.01 [0.01, 0.02] | 2011 |
| Ojurongbe et al (2011) | 0.01 | 0.01 | 4.8% | 0.01 [-0.01, 0.03] | 2011 |
| Sanyaolu et al (2011) | 0.015 | 0.015 | 3.5% | 0.01 [-0.01, 0.04] | 2011 |
| Udeh et al (2019) | 0.009 | 0.003 | 6.7% | 0.01 [0.00, 0.01] | 2019 |
| **Subtotal (95% CI)** | | | **25.2%** | **0.02 [0.00, 0.03]** | |
| Heterogeneity: Tau² = 0.00; Chi² = 20.96, df = 5 (P = 0.0008); I² = 76% | | | | | |
| Test for overall effect: Z = 2.80 (P = 0.005) | | | | | |
| **Total (95% CI)** | | | **100.0%** | **0.03 [0.02, 0.04]** | |
| Heterogeneity: Tau² = 0.00; Chi² = 292.57, df = 26 (P < 0.00001); I² = 91% | | | | | |
| Test for overall effect: Z = 7.87 (P < 0.00001) | | | | | |
| Test for subgroup differences: Chi² = 11.19, df = 3 (P = 0.01), I² = 73.2% | | | | | |

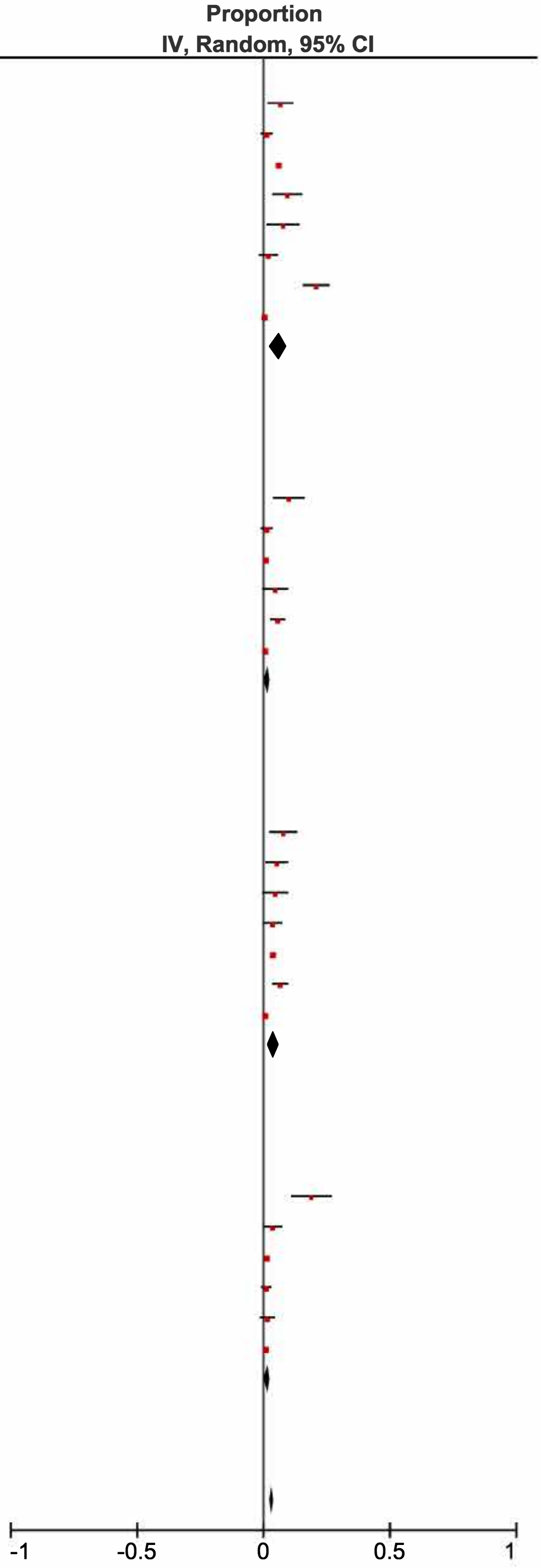


Supp. Figure 2

| Study or Subgroup | Proportion | SE | Weight | Proportion IV, Random, 95% CI | Year |
|---|---|---|---|---|---|
| **Pooled prevalence of AL** | | | | | |
| Hailemariam et al (2004) | 0.308 | 0.052 | 0.7% | 0.31 [0.21, 0.41] | 2004 |
| Tedesse & Kassu (2005) | 0.086 | 0.033 | 1.4% | 0.09 [0.02, 0.15] | 2005 |
| Moges et al (2006) | 0.286 | 0.085 | 0.3% | 0.29 [0.12, 0.45] | 2006 |
| Mariam et al (2008) | 0.211 | 0.039 | 1.1% | 0.21 [0.13, 0.29] | 2008 |
| Adamu & Petros (2009) | 0.025 | 0.011 | 2.7% | 0.03 [0.00, 0.05] | 2009 |
| Assefa et al (2009) | 0.121 | 0.022 | 2.0% | 0.12 [0.08, 0.16] | 2009 |
| Fekadu et al (2013) | 0.12 | 0.018 | 2.3% | 0.12 [0.08, 0.16] | 2013 |
| Teklemariam et al (2013) | 0.008 | 0.005 | 3.0% | 0.01 [-0.00, 0.02] | 2013 |
| Zeynudin et al (2013) | 0.143 | 0.037 | 1.2% | 0.14 [0.07, 0.22] | 2013 |
| Mengist et al (2015) | 0.05 | 0.016 | 2.4% | 0.05 [0.02, 0.08] | 2015 |
| Gedle et al (2017) | 0.031 | 0.01 | 2.8% | 0.03 [0.01, 0.05] | 2017 |
| Hailegebriel et al (2017) | 0.049 | 0.014 | 2.5% | 0.05 [0.02, 0.08] | 2017 |
| Eshetu et al (2017) | 0.067 | 0.017 | 2.3% | 0.07 [0.03, 0.10] | 2017 |
| Alemayehu et al (2020) | 0.06 | 0.12 | 0.2% | 0.06 [-0.18, 0.30] | 2020 |
| **Subtotal (95% CI)** | | | **24.9%** | **0.09 [0.06, 0.12]** | |
| Heterogeneity: Tau² = 0.00; Chi² = 140.49, df = 13 (P < 0.00001); I² = 91% | | | | | |
| Test for overall effect: Z = 5.98 (P < 0.00001) | | | | | |
| **Pooled prevalence of TT** | | | | | |
| Hailemariam et al (2004) | 0.064 | 0.028 | 1.6% | 0.06 [0.01, 0.12] | 2004 |
| Moges et al (2006) | 0.321 | 0.088 | 0.3% | 0.32 [0.15, 0.49] | 2006 |
| Mariam et al (2008) | 0.174 | 0.036 | 1.2% | 0.17 [0.10, 0.24] | 2008 |
| Assefa et al (2009) | 0.037 | 0.013 | 2.6% | 0.04 [0.01, 0.06] | 2009 |
| Zeynudin et al (2013) | 0.044 | 0.021 | 2.0% | 0.04 [0.00, 0.09] | 2013 |
| Fekadu et al (2013) | 0.009 | 0.005 | 3.0% | 0.01 [-0.00, 0.02] | 2013 |
| Hailegebriel et al (2017) | 0.018 | 0.009 | 2.8% | 0.02 [0.00, 0.04] | 2017 |
| Eshetu et al (2017) | 0.013 | 0.008 | 2.9% | 0.01 [-0.00, 0.03] | 2017 |
| Alemayehu et al (2020) | 0.016 | 0.006 | 3.0% | 0.02 [0.00, 0.03] | 2020 |
| **Subtotal (95% CI)** | | | **19.5%** | **0.03 [0.02, 0.05]** | |
| Heterogeneity: Tau² = 0.00; Chi² = 40.64, df = 8 (P < 0.00001); I² = 80% | | | | | |
| Test for overall effect: Z = 3.76 (P = 0.0002) | | | | | |
| **Pooled prevalence of HW** | | | | | |
| Hailemariam et al (2004) | 0.026 | 0.018 | 2.3% | 0.03 [-0.01, 0.06] | 2004 |
| Moges et al (2006) | 0.214 | 0.078 | 0.4% | 0.21 [0.06, 0.37] | 2006 |
| Mariam et al (2008) | 0.055 | 0.022 | 2.0% | 0.06 [0.01, 0.10] | 2008 |
| Assefa et al (2009) | 0.033 | 0.012 | 2.7% | 0.03 [0.01, 0.06] | 2009 |
| Fekadu et al (2013) | 0.026 | 0.009 | 2.8% | 0.03 [0.01, 0.04] | 2013 |
| Teklemariam et al (2013) | 0.019 | 0.007 | 2.9% | 0.02 [0.01, 0.03] | 2013 |
| Mengist et al (2015) | 0.044 | 0.015 | 2.5% | 0.04 [0.01, 0.07] | 2015 |
| Gedle et al (2017) | 0.006 | 0.004 | 3.1% | 0.01 [-0.00, 0.01] | 2017 |
| Hailegebriel et al (2017) | 0.022 | 0.01 | 2.8% | 0.02 [0.00, 0.04] | 2017 |
| Eshetu et al (2017) | 0.013 | 0.008 | 2.9% | 0.01 [-0.00, 0.03] | 2017 |
| Alemayehu et al (2020) | 0.01 | 0.005 | 3.0% | 0.01 [0.00, 0.02] | 2020 |
| **Subtotal (95% CI)** | | | **27.3%** | **0.02 [0.01, 0.03]** | |
| Heterogeneity: Tau² = 0.00; Chi² = 24.55, df = 10 (P = 0.006); I² = 59% | | | | | |
| Test for overall effect: Z = 4.62 (P < 0.00001) | | | | | |
| **Pooled prevalence of SS** | | | | | |
| Hailemariam et al (2004) | 0.051 | 0.025 | 1.8% | 0.05 [0.00, 0.10] | 2004 |
| Tedesse & Kassu (2005) | 0.171 | 0.045 | 0.9% | 0.17 [0.08, 0.26] | 2005 |
| Moges et al (2006) | 0.107 | 0.058 | 0.6% | 0.11 [-0.01, 0.22] | 2006 |
| Mariam et al (2008) | 0.064 | 0.023 | 1.9% | 0.06 [0.02, 0.11] | 2008 |
| Assefa et al (2009) | 0.126 | 0.023 | 1.9% | 0.13 [0.08, 0.17] | 2009 |
| Getaneh et al (2010) | 0.12 | 0.023 | 1.9% | 0.12 [0.07, 0.17] | 2010 |
| Fekadu et al (2013) | 0.163 | 0.02 | 2.1% | 0.16 [0.12, 0.20] | 2013 |
| Teklemariam et al (2013) | 0.04 | 0.01 | 2.8% | 0.04 [0.02, 0.06] | 2013 |
| Zeynudin et al (2013) | 0.066 | 0.026 | 1.7% | 0.07 [0.02, 0.12] | 2013 |
| Mengist et al (2015) | 0.05 | 0.016 | 2.4% | 0.05 [0.02, 0.08] | 2015 |
| Eshetu et al (2017) | 0.036 | 0.012 | 2.7% | 0.04 [0.01, 0.06] | 2017 |
| Gedle et al (2017) | 0.015 | 0.007 | 2.9% | 0.01 [0.00, 0.03] | 2017 |
| Hailegebriel et al (2017) | 0.173 | 0.025 | 1.8% | 0.17 [0.12, 0.22] | 2017 |
| Alemayehu et al (2020) | 0.039 | 0.01 | 2.8% | 0.04 [0.02, 0.06] | 2020 |
| **Subtotal (95% CI)** | | | **28.3%** | **0.08 [0.05, 0.11]** | |
| Heterogeneity: Tau² = 0.00; Chi² = 113.54, df = 13 (P < 0.00001); I² = 89% | | | | | |
| Test for overall effect: Z = 6.17 (P < 0.00001) | | | | | |
| **Total (95% CI)** | | | **100.0%** | **0.06 [0.05, 0.07]** | |
| Heterogeneity: Tau² = 0.00; Chi² = 384.27, df = 47 (P < 0.00001); I² = 88% | | | | | |
| Test for overall effect: Z = 10.89 (P < 0.00001) | | | | | |
| Test for subgroup differences: Chi² = 35.36, df = 3 (P < 0.00001), I² = 91.5% | | | | | |

Proportion
IV, Random, 95% CI

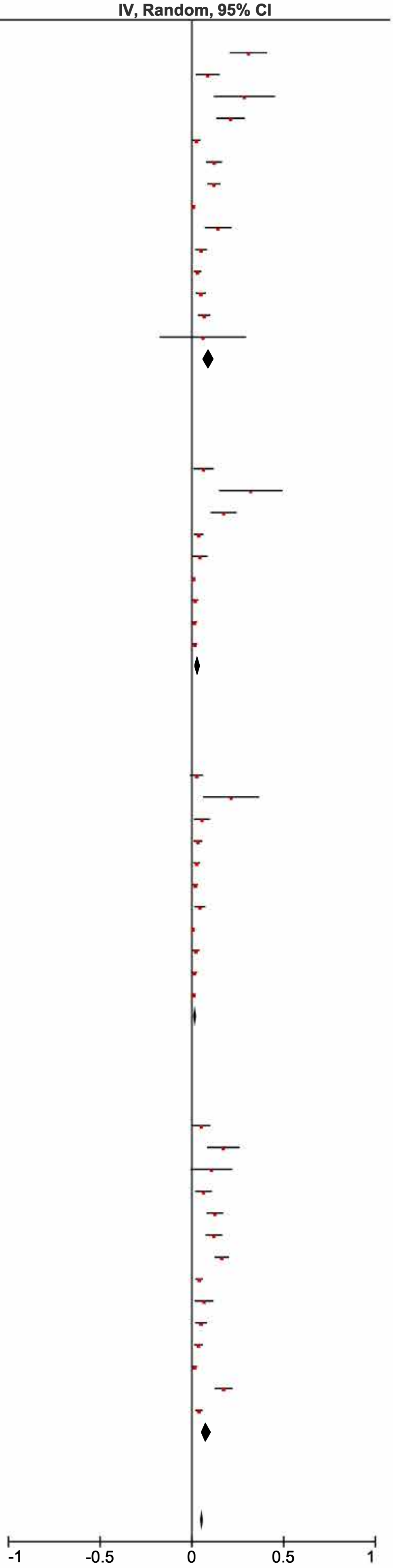


Supp. Figure 3

| Study or Subgroup | Proportion | SE | Weight | Proportion IV, Random, 95% CI |
|---|---|---|---|---|
| **Pooled prevalence of AL** | | | | |
| Cerveja et al (2017) | 0.04 | 0.01 | 24.7% | 0.04 [0.02, 0.06] |
| **Subtotal (95% CI)** | | | **24.7%** | **0.04 [0.02, 0.06]** |
| Heterogeneity: Not applicable | | | | |
| Test for overall effect: Z = 4.00 (P < 0.0001) | | | | |
| **Pooled prevalence of TT** | | | | |
| Cerveja et al (2017) | 0.111 | 0.016 | 21.0% | 0.11 [0.08, 0.14] |
| **Subtotal (95% CI)** | | | **21.0%** | **0.11 [0.08, 0.14]** |
| Heterogeneity: Not applicable | | | | |
| Test for overall effect: Z = 6.94 (P < 0.00001) | | | | |
| **Pooled prevalence of HW** | | | | |
| Cerveja et al (2017) | 0.003 | 0.003 | 27.6% | 0.00 [-0.00, 0.01] |
| **Subtotal (95% CI)** | | | **27.6%** | **0.00 [-0.00, 0.01]** |
| Heterogeneity: Not applicable | | | | |
| Test for overall effect: Z = 1.00 (P = 0.32) | | | | |
| **Pooled prevalence of SS** | | | | |
| Cerveja et al (2017) | 0.013 | 0.006 | 26.7% | 0.01 [0.00, 0.02] |
| **Subtotal (95% CI)** | | | **26.7%** | **0.01 [0.00, 0.02]** |
| Heterogeneity: Not applicable | | | | |
| Test for overall effect: Z = 2.17 (P = 0.03) | | | | |
| **Total (95% CI)** | | | **100.0%** | **0.04 [0.01, 0.07]** |

Heterogeneity: Tau² = 0.00; Chi² = 54.54, df = 3 (P < 0.00001); I² = 94%

Test for overall effect: Z = 2.54 (P = 0.01)

Test for subgroup differences: Chi² = 54.54, df = 3 (P < 0.00001), I² = 94.5%

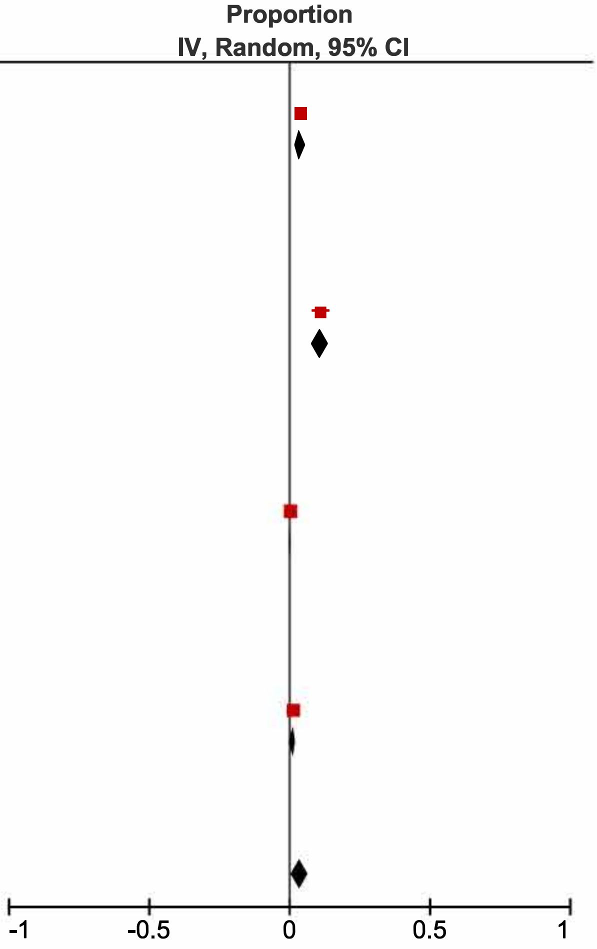


Supp. Figure 4

| Study or Subgroup | Proportion | SE | Weight | Proportion IV, Random, 95% CI |
|---|---|---|---|---|
| **Pooled prevalence of AL** | | | | |
| Adeleke et al (2015) | 0.1 | 0.02 | 15.0% | 0.10 [0.06, 0.14] |
| **Subtotal (95% CI)** | | | **15.0%** | **0.10 [0.06, 0.14]** |
| Heterogeneity: Not applicable | | | | |
| Test for overall effect: Z = 5.00 (P < 0.00001) | | | | |
| **Pooled prevalence of TT** | | | | |
| Adeleke et al (2015) | 0.013 | 0.007 | 28.3% | 0.01 [-0.00, 0.03] |
| **Subtotal (95% CI)** | | | **28.3%** | **0.01 [-0.00, 0.03]** |
| Heterogeneity: Not applicable | | | | |
| Test for overall effect: Z = 1.86 (P = 0.06) | | | | |
| **Pooled prevalence of HW** | | | | |
| Adeleke et al (2015) | 0.013 | 0.007 | 28.3% | 0.01 [-0.00, 0.03] |
| **Subtotal (95% CI)** | | | **28.3%** | **0.01 [-0.00, 0.03]** |
| Heterogeneity: Not applicable | | | | |
| Test for overall effect: Z = 1.86 (P = 0.06) | | | | |
| **Pooled prevalence of SS** | | | | |
| Adeleke et al (2015) | 0.013 | 0.007 | 28.3% | 0.01 [-0.00, 0.03] |
| **Subtotal (95% CI)** | | | **28.3%** | **0.01 [-0.00, 0.03]** |
| Heterogeneity: Not applicable | | | | |
| Test for overall effect: Z = 1.86 (P = 0.06) | | | | |
| **Total (95% CI)** | | | **100.0%** | **0.03 [0.01, 0.05]** |

Heterogeneity: $Tau^2 = 0.00$; $Chi^2 = 18.18$, df = 3 (P = 0.0004); $I^2 = 83\%$
Test for overall effect: Z = 2.46 (P = 0.01)
Test for subgroup differences: $Chi^2 = 18.18$, df = 3 (P = 0.0004), $I^2 = 83.5\%$

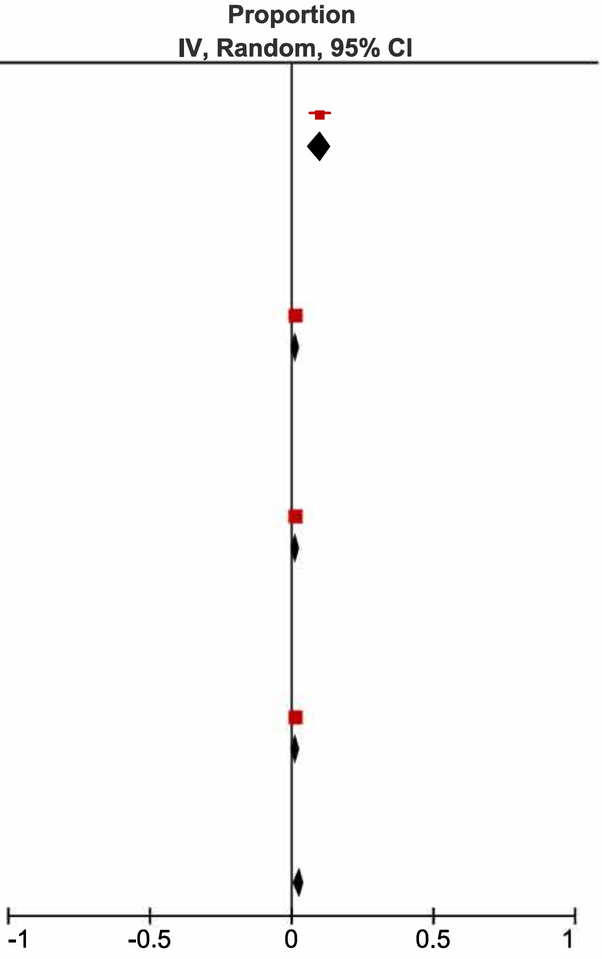


Supp. Figure 5

| Study or Subgroup | Proportion | SE | Weight | Proportion IV, Random, 95% CI |
|---|---|---|---|---|
| **Pooled prevalence of AL** | | | | |
| Dowling et al (2002) | 0.023 | 0.1 | 6.9% | 0.02 [-0.17, 0.22] |
| Hoesseinipour et al (2007) | 0.03 | 0.1 | 6.9% | 0.03 [-0.17, 0.23] |
| **Subtotal (95% CI)** | | | **13.8%** | **0.03 [-0.11, 0.17]** |
| Heterogeneity: Tau² = 0.00; Chi² = 0.00, df = 1 (P = 0.96); I² = 0% | | | | |
| Test for overall effect: Z = 0.37 (P = 0.71) | | | | |
| **Pooled prevalence of HW** | | | | |
| Dowling et al (2002) | 0.192 | 0.027 | 19.8% | 0.19 [0.14, 0.24] |
| Hoesseinipour et al (2007) | 0.102 | 0.019 | 21.4% | 0.10 [0.06, 0.14] |
| **Subtotal (95% CI)** | | | **41.1%** | **0.14 [0.06, 0.23]** |
| Heterogeneity: Tau² = 0.00; Chi² = 7.43, df = 1 (P = 0.006); I² = 87% | | | | |
| Test for overall effect: Z = 3.22 (P = 0.001) | | | | |
| **Pooled prevalence of SS** | | | | |
| Dowling et al (2002) | 0.055 | 0.015 | 22.0% | 0.06 [0.03, 0.08] |
| Hoesseinipour et al (2007) | 0.008 | 0.005 | 23.1% | 0.01 [-0.00, 0.02] |
| **Subtotal (95% CI)** | | | **45.1%** | **0.03 [-0.02, 0.08]** |
| Heterogeneity: Tau² = 0.00; Chi² = 8.84, df = 1 (P = 0.003); I² = 89% | | | | |
| Test for overall effect: Z = 1.26 (P = 0.21) | | | | |
| **Total (95% CI)** | | | **100.0%** | **0.08 [0.02, 0.14]** |
| Heterogeneity: Tau² = 0.00; Chi² = 70.02, df = 5 (P < 0.00001); I² = 93% | | | | |
| Test for overall effect: Z = 2.47 (P = 0.01) | | | | |
| Test for subgroup differences: Chi² = 5.34, df = 2 (P = 0.07), I² = 62.6% | | | | |

Proportion
IV, Random, 95% CI

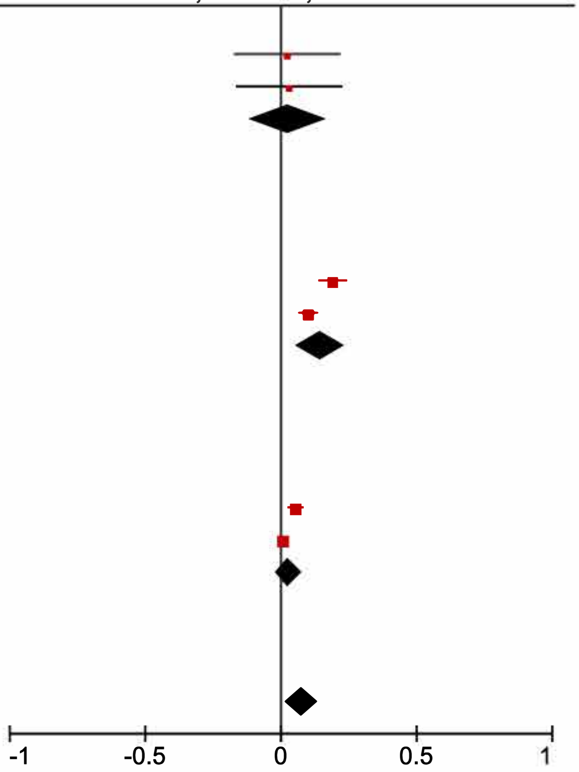


Supp. Figure 6

| Study or Subgroup | Proportion | SE | Weight | Proportion IV, Random, 95% CI | Year |
|---|---|---|---|---|---|
| **Pooled prevalence of AL** | | | | | |
| Idindili et al (2011) | 0.128 | 0.016 | 22.5% | 0.13 [0.10, 0.16] | 2011 |
| **Subtotal (95% CI)** | | | **22.5%** | **0.13 [0.10, 0.16]** | |
| Heterogeneity: Not applicable | | | | | |
| Test for overall effect: Z = 8.00 (P < 0.00001) | | | | | |
| **Pooled prevalence of HW** | | | | | |
| Mwambete et al (2010) | 0.152 | 0.044 | 12.7% | 0.15 [0.07, 0.24] | 2010 |
| Idindili et al (2011) | 0.154 | 0.018 | 21.8% | 0.15 [0.12, 0.19] | 2011 |
| **Subtotal (95% CI)** | | | **34.6%** | **0.15 [0.12, 0.19]** | |
| Heterogeneity: Tau² = 0.00; Chi² = 0.00, df = 1 (P = 0.97); I² = 0% | | | | | |
| Test for overall effect: Z = 9.23 (P < 0.00001) | | | | | |
| **Pooled prevalence of SS** | | | | | |
| Mwambete et al (2010) | 0.03 | 0.021 | 20.8% | 0.03 [-0.01, 0.07] | 2010 |
| Idindili et al (2011) | 0.135 | 0.017 | 22.2% | 0.14 [0.10, 0.17] | 2011 |
| **Subtotal (95% CI)** | | | **42.9%** | **0.08 [-0.02, 0.19]** | |
| Heterogeneity: Tau² = 0.01; Chi² = 15.10, df = 1 (P = 0.0001); I² = 93% | | | | | |
| Test for overall effect: Z = 1.59 (P = 0.11) | | | | | |
| **Total (95% CI)** | | | **100.0%** | **0.12 [0.07, 0.16]** | |
| Heterogeneity: Tau² = 0.00; Chi² = 23.49, df = 4 (P = 0.0001); I² = 83% | | | | | |
| Test for overall effect: Z = 5.31 (P < 0.00001) | | | | | |
| Test for subgroup differences: Chi² = 2.37, df = 2 (P = 0.31), I² = 15.6% | | | | | |

Proportion IV, Random, 95% CI

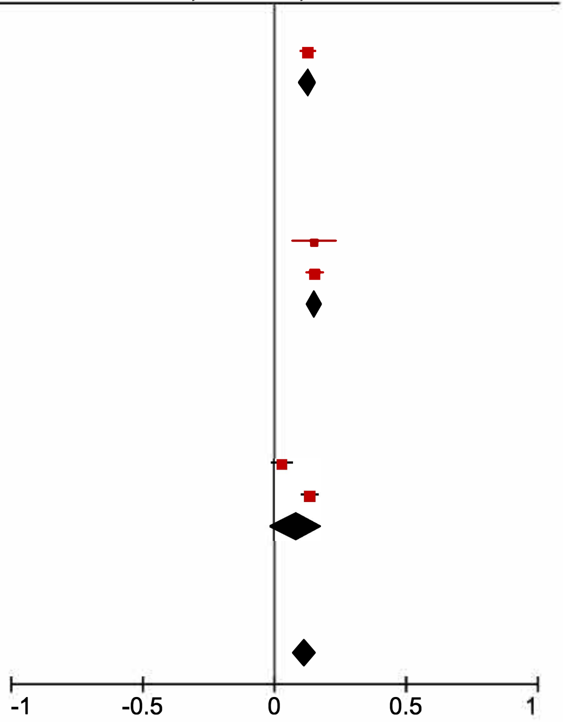


Supp. Figure 7

| Study or Subgroup | Proportion | SE | Weight | Proportion IV, Random, 95% CI |
|---|---|---|---|---|
| **Pooled prevalence of HW** | | | | |
| Lebbad et al (2001) | 0.108 | 0.051 | 54.7% | 0.11 [0.01, 0.21] |
| **Subtotal (95% CI)** | | | **54.7%** | **0.11 [0.01, 0.21]** |
| Heterogeneity: Not applicable | | | | |
| Test for overall effect: Z = 2.12 (P = 0.03) | | | | |
| **Pooled prevalence of SS** | | | | |
| Lebbad et al (2001) | 0.135 | 0.056 | 45.3% | 0.14 [0.03, 0.24] |
| **Subtotal (95% CI)** | | | **45.3%** | **0.14 [0.03, 0.24]** |
| Heterogeneity: Not applicable | | | | |
| Test for overall effect: Z = 2.41 (P = 0.02) | | | | |
| **Total (95% CI)** | | | **100.0%** | **0.12 [0.05, 0.19]** |
| Heterogeneity: Tau² = 0.00; Chi² = 0.13, df = 1 (P = 0.72); I² = 0% | | | | |
| Test for overall effect: Z = 3.19 (P = 0.001) | | | | |
| Test for subgroup differences: Chi² = 0.13, df = 1 (P = 0.72), I² = 0% | | | | |

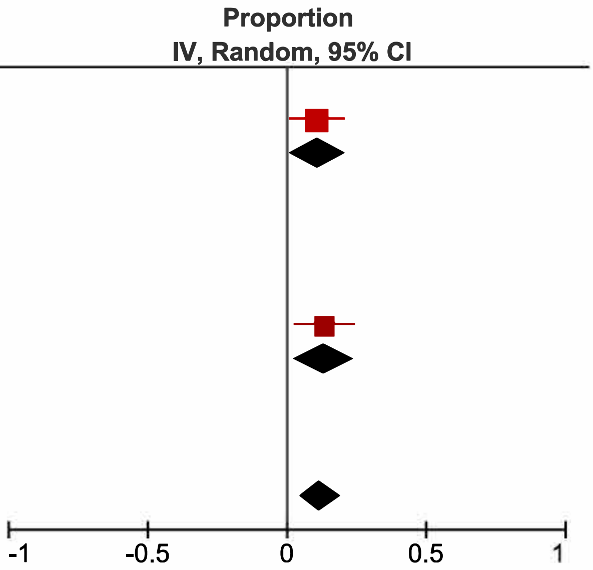


Supp. Figure 8

| Study or Subgroup | Proportion | SE | Weight | Proportion IV, Random, 95% CI |
|---|---|---|---|---|
| **Pooled prevalence of AL** | | | | |
| Roka et al (2012) | 0.192 | 0.024 | 12.4% | 0.19 [0.14, 0.24] |
| Roka et al (2013) | 0.231 | 0.025 | 12.4% | 0.23 [0.18, 0.28] |
| **Subtotal (95% CI)** | | | **24.9%** | **0.21 [0.17, 0.25]** |
| Heterogeneity: Tau² = 0.00; Chi² = 1.27, df = 1 (P = 0.26); I² = 21% | | | | |
| Test for overall effect: Z = 10.82 (P < 0.00001) | | | | |
| **Pooled prevalence of TT** | | | | |
| Roka et al (2012) | 0.515 | 0.031 | 12.2% | 0.52 [0.45, 0.58] |
| Roka et al (2013) | 0.454 | 0.03 | 12.2% | 0.45 [0.40, 0.51] |
| **Subtotal (95% CI)** | | | **24.5%** | **0.48 [0.42, 0.54]** |
| Heterogeneity: Tau² = 0.00; Chi² = 2.00, df = 1 (P = 0.16); I² = 50% | | | | |
| Test for overall effect: Z = 15.87 (P < 0.00001) | | | | |
| **Pooled prevalence of HW** | | | | |
| Roka et al (2012) | 0.042 | 0.012 | 12.7% | 0.04 [0.02, 0.07] |
| Roka et al (2013) | 0.04 | 0.012 | 12.7% | 0.04 [0.02, 0.06] |
| **Subtotal (95% CI)** | | | **25.5%** | **0.04 [0.02, 0.06]** |
| Heterogeneity: Tau² = 0.00; Chi² = 0.01, df = 1 (P = 0.91); I² = 0% | | | | |
| Test for overall effect: Z = 4.83 (P < 0.00001) | | | | |
| **Pooled prevalence of SS** | | | | |
| Roka et al (2012) | 0.088 | 0.018 | 12.6% | 0.09 [0.05, 0.12] |
| Roka et al (2013) | 0.103 | 0.018 | 12.6% | 0.10 [0.07, 0.14] |
| **Subtotal (95% CI)** | | | **25.2%** | **0.10 [0.07, 0.12]** |
| Heterogeneity: Tau² = 0.00; Chi² = 0.35, df = 1 (P = 0.56); I² = 0% | | | | |
| Test for overall effect: Z = 7.50 (P < 0.00001) | | | | |
| **Total (95% CI)** | | | **100.0%** | **0.21 [0.11, 0.30]** |
| Heterogeneity: Tau² = 0.02; Chi² = 404.72, df = 7 (P < 0.00001); I² = 98% | | | | |
| Test for overall effect: Z = 4.14 (P < 0.0001) | | | | |
| Test for subgroup differences: Chi² = 238.53, df = 3 (P < 0.00001), I² = 98.7% | | | | |

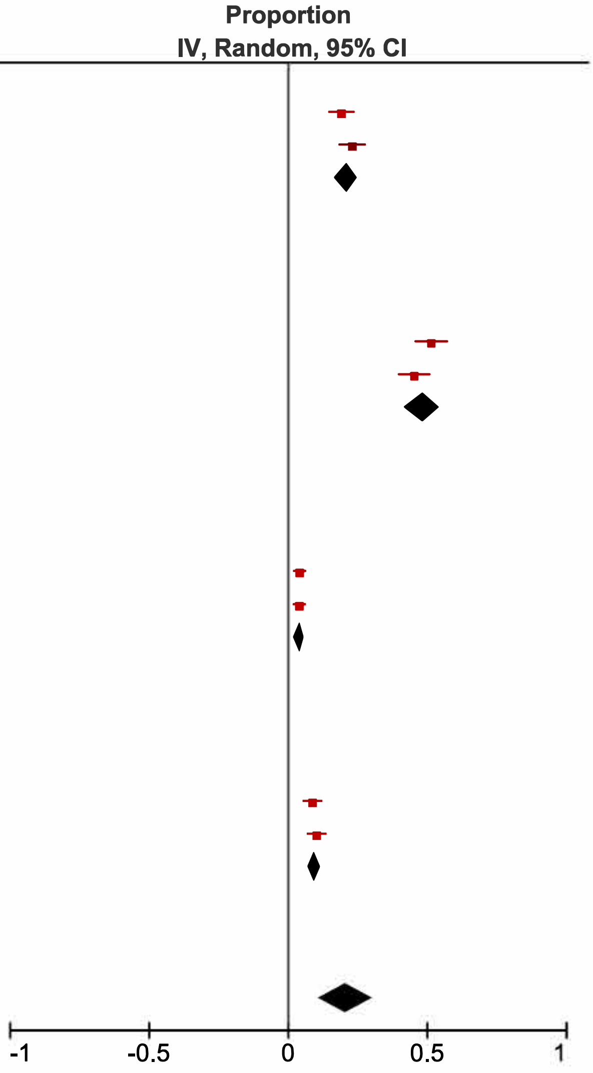


Supp. Figure 9

| Study or Subgroup | Proportion | SE | Weight | Proportion IV, Fixed, 95% CI | Year |
|---|---|---|---|---|---|
| **Pooled prevalence of AL** | | | | | |
| Walson et al (2010) | 0.033 | 0.005 | 3.2% | 0.03 [0.02, 0.04] | 2010 |
| Kipyegen et al (2012) | 0.046 | 0.012 | 0.6% | 0.05 [0.02, 0.07] | 2012 |
| Arndt et al (2013) | 0.039 | 0.016 | 0.3% | 0.04 [0.01, 0.07] | 2013 |
| **Subtotal (95% CI)** | | | **4.1%** | **0.04 [0.03, 0.04]** | |
| Heterogeneity: Chi² = 1.06, df = 2 (P = 0.59); I² = 0% | | | | | |
| Test for overall effect: Z = 7.95 (P < 0.00001) | | | | | |
| **Pooled prevalence of TT** | | | | | |
| Walson et al (2010) | 0.017 | 0.003 | 8.9% | 0.02 [0.01, 0.02] | 2010 |
| Kipyegen et al (2012) | 0.011 | 0.006 | 2.2% | 0.01 [-0.00, 0.02] | 2012 |
| **Subtotal (95% CI)** | | | **11.1%** | **0.02 [0.01, 0.02]** | |
| Heterogeneity: Chi² = 0.80, df = 1 (P = 0.37); I² = 0% | | | | | |
| Test for overall effect: Z = 5.89 (P < 0.00001) | | | | | |
| **Pooled prevalence of HW** | | | | | |
| Walson et al (2010) | 0.109 | 0.008 | 1.2% | 0.11 [0.09, 0.12] | 2010 |
| Kipyegen et al (2012) | 0.007 | 0.005 | 3.2% | 0.01 [-0.00, 0.02] | 2012 |
| Arndt et al (2013) | 0.203 | 0.032 | 0.1% | 0.20 [0.14, 0.27] | 2013 |
| **Subtotal (95% CI)** | | | **4.5%** | **0.04 [0.03, 0.05]** | |
| Heterogeneity: Chi² = 143.78, df = 2 (P < 0.00001); I² = 99% | | | | | |
| Test for overall effect: Z = 9.17 (P < 0.00001) | | | | | |
| **Pooled prevalence of SS** | | | | | |
| Walson et al (2010) | 0.003 | 0.001 | 79.9% | 0.00 [0.00, 0.00] | 2010 |
| Arndt et al (2013) | 0.033 | 0.014 | 0.4% | 0.03 [0.01, 0.06] | 2013 |
| **Subtotal (95% CI)** | | | **80.3%** | **0.00 [0.00, 0.01]** | |
| Heterogeneity: Chi² = 4.57, df = 1 (P = 0.03); I² = 78% | | | | | |
| Test for overall effect: Z = 3.16 (P = 0.002) | | | | | |
| **Total (95% CI)** | | | **100.0%** | **0.01 [0.01, 0.01]** | |
| Heterogeneity: Chi² = 272.42, df = 9 (P < 0.00001); I² = 97% | | | | | |
| Test for overall effect: Z = 8.35 (P < 0.00001) | | | | | |
| Test for subgroup differences: Chi² = 122.22, df = 3 (P < 0.00001), I² = 97.5% | | | | | |

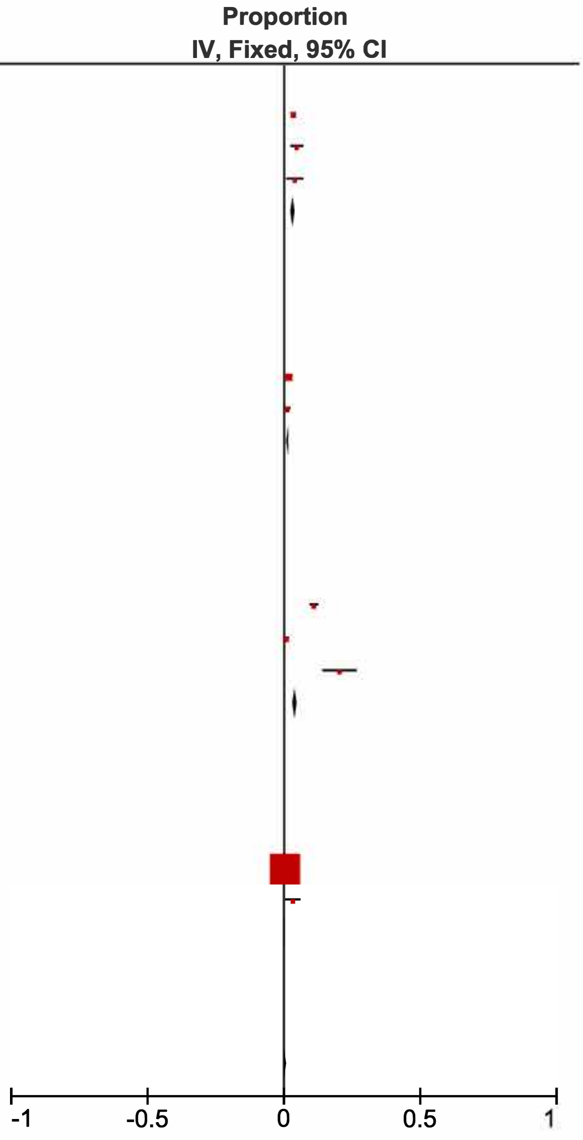


Supp. Figure 10

| Study or Subgroup | Proportion | SE | Weight | Proportion IV, Fixed, 95% CI |
|---|---|---|---|---|
| **Pooled prevalence of AL** | | | | |
| Chintu et al (1995) | 0.091 | 0.043 | 2.0% | 0.09 [0.01, 0.18] |
| Modjarrad et al (2005) | 0.131 | 0.02 | 9.3% | 0.13 [0.09, 0.17] |
| **Subtotal (95% CI)** | | | **11.3%** | **0.12 [0.09, 0.16]** |
| Heterogeneity: Chi² = 0.71, df = 1 (P = 0.40); I² = 0% | | | | |
| Test for overall effect: Z = 6.83 (P < 0.00001) | | | | |
| **Pooled prevalence of HW** | | | | |
| Modjarrad et al (2005) | 0.098 | 0.017 | 12.9% | 0.10 [0.06, 0.13] |
| **Subtotal (95% CI)** | | | **12.9%** | **0.10 [0.06, 0.13]** |
| Heterogeneity: Not applicable | | | | |
| Test for overall effect: Z = 5.76 (P < 0.00001) | | | | |
| **Pooled prevalence of SS** | | | | |
| Modjarrad et al (2005) | 0.017 | 0.007 | 75.8% | 0.02 [0.00, 0.03] |
| **Subtotal (95% CI)** | | | **75.8%** | **0.02 [0.00, 0.03]** |
| Heterogeneity: Not applicable | | | | |
| Test for overall effect: Z = 2.43 (P = 0.02) | | | | |
| **Total (95% CI)** | | | **100.0%** | **0.04 [0.03, 0.05]** |
| Heterogeneity: Chi² = 44.54, df = 3 (P < 0.00001); I² = 93% | | | | |
| Test for overall effect: Z = 6.48 (P < 0.00001) | | | | |
| Test for subgroup differences: Chi² = 43.83, df = 2 (P < 0.00001), I² = 95.4% | | | | |

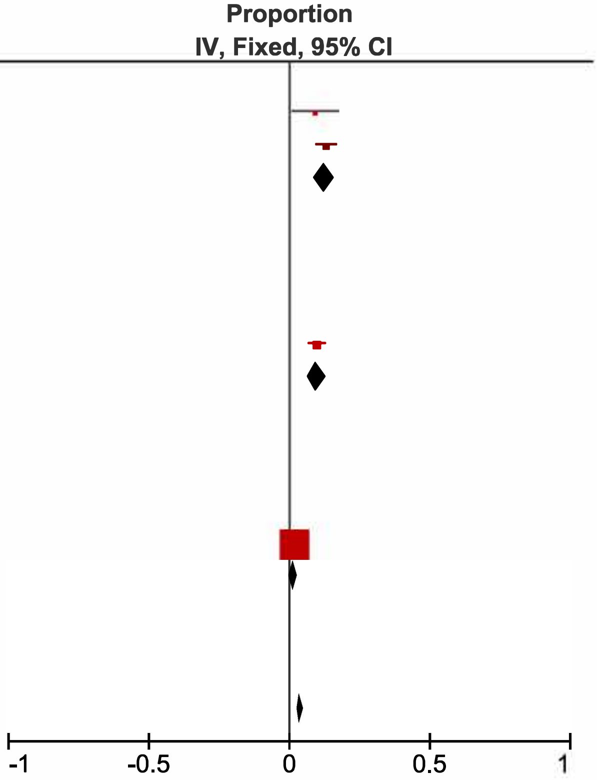


Supp. Figure 11

| Study or Subgroup | Proportion | SE | Weight | Proportion IV, Random, 95% CI |
|---|---|---|---|---|
| **Pooled prevalence of TT** | | | | |
| Morawski et al (2017) | 0.005 | 0.005 | 35.0% | 0.01 [-0.00, 0.01] |
| **Subtotal (95% CI)** | | | **35.0%** | **0.01 [-0.00, 0.01]** |
| Heterogeneity: Not applicable | | | | |
| Test for overall effect: Z = 1.00 (P = 0.32) | | | | |
| **Pooled prevalence of HW** | | | | |
| Morawski et al (2017) | 0.277 | 0.031 | 30.9% | 0.28 [0.22, 0.34] |
| **Subtotal (95% CI)** | | | **30.9%** | **0.28 [0.22, 0.34]** |
| Heterogeneity: Not applicable | | | | |
| Test for overall effect: Z = 8.94 (P < 0.00001) | | | | |
| **Pooled prevalence of SS** | | | | |
| Morawski et al (2017) | 0.04 | 0.014 | 34.1% | 0.04 [0.01, 0.07] |
| **Subtotal (95% CI)** | | | **34.1%** | **0.04 [0.01, 0.07]** |
| Heterogeneity: Not applicable | | | | |
| Test for overall effect: Z = 2.86 (P = 0.004) | | | | |
| **Total (95% CI)** | | | **100.0%** | **0.10 [0.00, 0.20]** |

Heterogeneity: Tau² = 0.01; Chi² = 78.62, df = 2 (P < 0.00001); I² = 97%
Test for overall effect: Z = 2.03 (P = 0.04)
Test for subgroup differences: Chi² = 78.62, df = 2 (P < 0.00001), I² = 97.5%

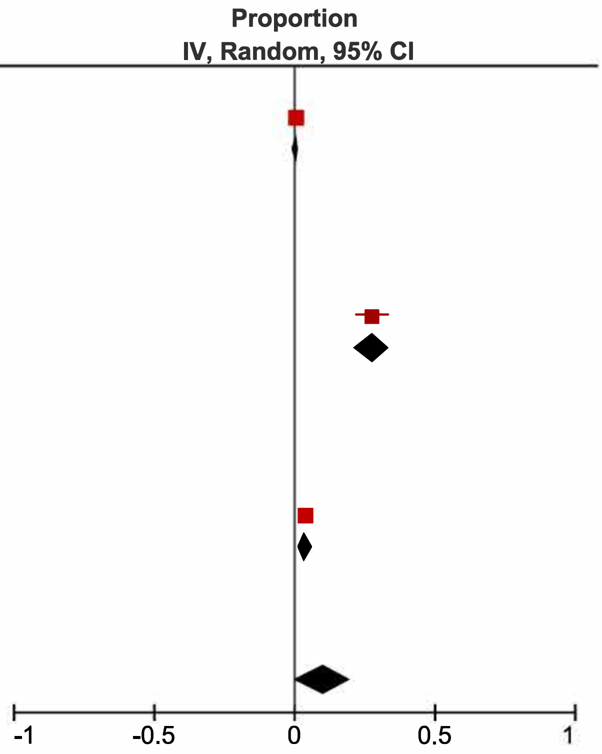


Supp. Figure 12

| Study or Subgroup | Proportion | SE | Weight | Proportion IV, Random, 95% CI |
|---|---|---|---|---|
| **Pooled prevalence of AL** | | | | |
| Vouking et al (2014) | 0.13 | 0.023 | 14.1% | 0.13 [0.08, 0.18] |
| **Subtotal (95% CI)** | | | **14.1%** | **0.13 [0.08, 0.18]** |
| Heterogeneity: Not applicable | | | | |
| Test for overall effect: Z = 5.65 (P < 0.00001) | | | | |
| **Pooled prevalence of TT** | | | | |
| Nkenfou et al (2013) | 0.024 | 0.024 | 13.6% | 0.02 [-0.02, 0.07] |
| Vouking et al (2014) | 0.043 | 0.014 | 18.5% | 0.04 [0.02, 0.07] |
| **Subtotal (95% CI)** | | | **32.1%** | **0.04 [0.01, 0.06]** |
| Heterogeneity: Tau² = 0.00; Chi² = 0.47, df = 1 (P = 0.49); I² = 0% | | | | |
| Test for overall effect: Z = 3.16 (P = 0.002) | | | | |
| **Pooled prevalence of HW** | | | | |
| Vouking et al (2014) | 0.014 | 0.008 | 21.2% | 0.01 [-0.00, 0.03] |
| **Subtotal (95% CI)** | | | **21.2%** | **0.01 [-0.00, 0.03]** |
| Heterogeneity: Not applicable | | | | |
| Test for overall effect: Z = 1.75 (P = 0.08) | | | | |
| **Pooled prevalence of SS** | | | | |
| Nkenfou et al (2013) | 0.024 | 0.024 | 13.6% | 0.02 [-0.02, 0.07] |
| Vouking et al (2014) | 0.034 | 0.013 | 19.0% | 0.03 [0.01, 0.06] |
| **Subtotal (95% CI)** | | | **32.6%** | **0.03 [0.01, 0.05]** |
| Heterogeneity: Tau² = 0.00; Chi² = 0.13, df = 1 (P = 0.71); I² = 0% | | | | |
| Test for overall effect: Z = 2.78 (P = 0.006) | | | | |
| **Total (95% CI)** | | | **100.0%** | **0.04 [0.01, 0.07]** |
| Heterogeneity: Tau² = 0.00; Chi² = 23.98, df = 5 (P = 0.0002); I² = 79% | | | | |
| Test for overall effect: Z = 3.02 (P = 0.002) | | | | |
| Test for subgroup differences: Chi² = 23.38, df = 3 (P < 0.0001), I² = 87.2% | | | | |

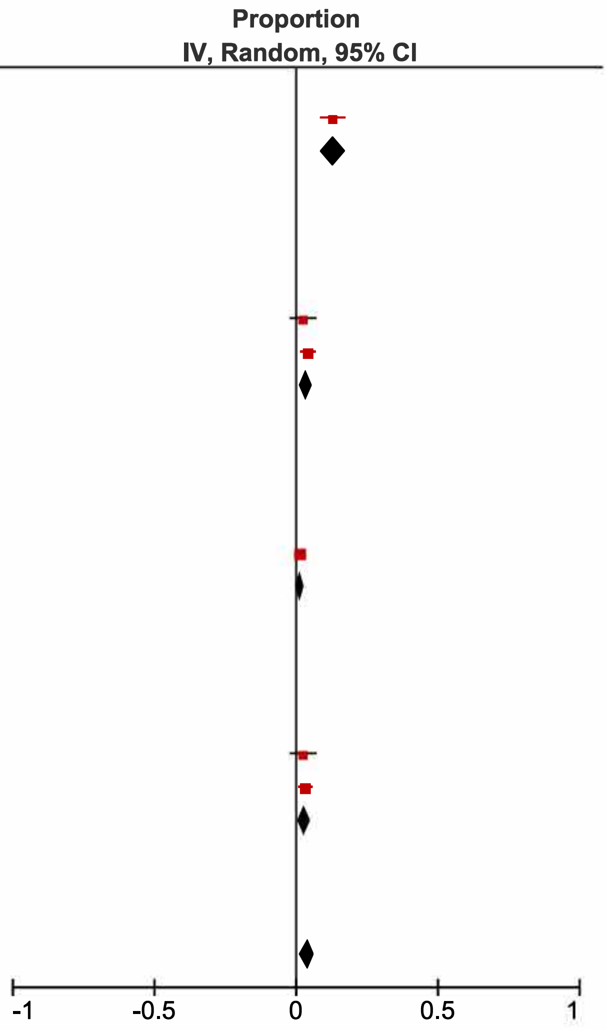


Supp. Figure 13

| Study or Subgroup | Proportion | SE | Weight | Proportion IV, Random, 95% CI |
|---|---|---|---|---|
| **Pooled prevalence of AL** | | | | |
| Wumba et al (2010) | 0.04 | 0.015 | 18.2% | 0.04 [0.01, 0.07] |
| **Subtotal (95% CI)** | | | **18.2%** | **0.04 [0.01, 0.07]** |
| Heterogeneity: Not applicable | | | | |
| Test for overall effect: Z = 2.67 (P = 0.008) | | | | |
| **Pooled prevalence of TT** | | | | |
| Wumba et al (2010) | 0.006 | 0.006 | 44.9% | 0.01 [-0.01, 0.02] |
| **Subtotal (95% CI)** | | | **44.9%** | **0.01 [-0.01, 0.02]** |
| Heterogeneity: Not applicable | | | | |
| Test for overall effect: Z = 1.00 (P = 0.32) | | | | |
| **Pooled prevalence of HW** | | | | |
| Wumba et al (2010) | 0.011 | 0.008 | 36.9% | 0.01 [-0.00, 0.03] |
| **Subtotal (95% CI)** | | | **36.9%** | **0.01 [-0.00, 0.03]** |
| Heterogeneity: Not applicable | | | | |
| Test for overall effect: Z = 1.38 (P = 0.17) | | | | |
| **Total (95% CI)** | | | **100.0%** | **0.01 [-0.00, 0.03]** |

Heterogeneity: Tau² = 0.00; Chi² = 4.43, df = 2 (P = 0.11); I² = 55%
Test for overall effect: Z = 1.85 (P = 0.06)
Test for subgroup differences: Chi² = 4.43, df = 2 (P = 0.11), I² = 54.9%

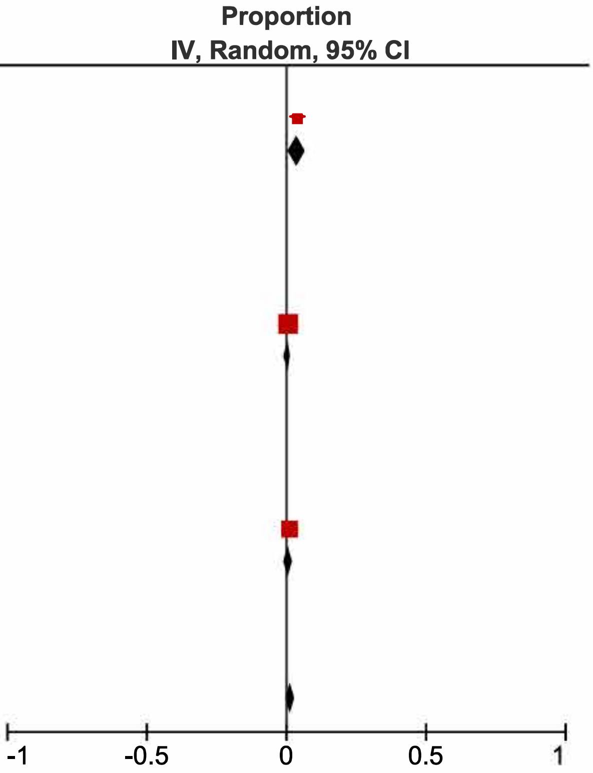


Supp. Figure 14

| Study or Subgroup | Proportion | SE | Weight | Proportion IV, Random, 95% CI | Proportion IV, Random, 95% CI |
|---|---|---|---|---|---|
| **Pooled prevalence of AL** | | | | | |
| Escobado & Nunez (1999) | 0.03 | 0.021 | 100.0% | 0.03 [-0.01, 0.07] | |
| **Subtotal (95% CI)** | | | **100.0%** | **0.03 [-0.01, 0.07]** | |
| Heterogeneity: Not applicable | | | | | |
| Test for overall effect: Z = 1.43 (P = 0.15) | | | | | |
| | | | | | |
| **Total (95% CI)** | | | **100.0%** | **0.03 [-0.01, 0.07]** | |
| Heterogeneity: Not applicable | | | | | |
| Test for overall effect: Z = 1.43 (P = 0.15) | | | | | |
| Test for subgroup differences: Not applicable | | | | | |

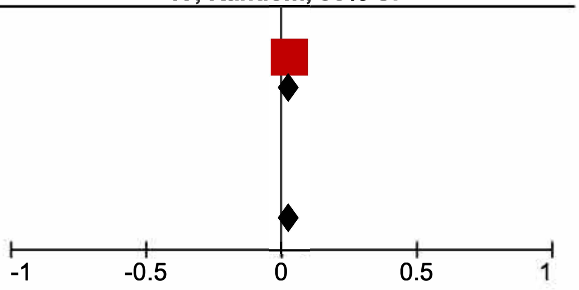


Supp. Figure 15

| Study or Subgroup | Proportion | SE | Weight | Proportion IV, Random, 95% CI | Year |
|---|---|---|---|---|---|
| **Pooled prevalence of AL** | | | | | |
| Cimerman et al (1999) | 0.025 | 0.011 | 6.3% | 0.03 [0.00, 0.05] | 1999 |
| Feitosa et al (2001) | 0.118 | 0.017 | 5.8% | 0.12 [0.08, 0.15] | 2001 |
| Marchiblatt & Cantos (2003) | 0.024 | 0.01 | 6.4% | 0.02 [0.00, 0.04] | 2003 |
| Rodrigues Bachur (2008) | 0.132 | 0.014 | 6.1% | 0.13 [0.10, 0.16] | 2008 |
| Amancio et al (2012) | 0.01 | 0.009 | 6.5% | 0.01 [-0.01, 0.03] | 2012 |
| **Subtotal (95% CI)** | | | **31.1%** | **0.06 [0.02, 0.11]** | |
| Heterogeneity: Tau² = 0.00; Chi² = 79.58, df = 4 (P < 0.00001); I² = 95% | | | | | |
| Test for overall effect: Z = 2.62 (P = 0.009) | | | | | |
| **Pooled prevalence of TT** | | | | | |
| Feitosa et al (2001) | 0.052 | 0.012 | 6.3% | 0.05 [0.03, 0.08] | 2001 |
| Marchiblatt & Cantos (2003) | 0.005 | 0.005 | 6.7% | 0.01 [-0.00, 0.01] | 2003 |
| Rodrigues Bachur (2008) | 0.11 | 0.013 | 6.2% | 0.11 [0.08, 0.14] | 2008 |
| **Subtotal (95% CI)** | | | **19.2%** | **0.05 [-0.01, 0.12]** | |
| Heterogeneity: Tau² = 0.00; Chi² = 63.59, df = 2 (P < 0.00001); I² = 97% | | | | | |
| Test for overall effect: Z = 1.72 (P = 0.08) | | | | | |
| **Pooled prevalence of HW** | | | | | |
| Feitosa et al (2001) | 0.044 | 0.011 | 6.3% | 0.04 [0.02, 0.07] | 2001 |
| Rodrigues Bachur (2008) | 0.117 | 0.013 | 6.2% | 0.12 [0.09, 0.14] | 2008 |
| Cardoso et al (2011) | 0.004 | 0.003 | 6.8% | 0.00 [-0.00, 0.01] | 2011 |
| **Subtotal (95% CI)** | | | **19.3%** | **0.05 [-0.01, 0.12]** | |
| Heterogeneity: Tau² = 0.00; Chi² = 80.81, df = 2 (P < 0.00001); I² = 98% | | | | | |
| Test for overall effect: Z = 1.67 (P = 0.09) | | | | | |
| **Pooled prevalence of SS** | | | | | |
| Cimerman et al (1999) | 0.025 | 0.011 | 6.3% | 0.03 [0.00, 0.05] | 1999 |
| Feitosa et al (2001) | 0.055 | 0.012 | 6.3% | 0.06 [0.03, 0.08] | 2001 |
| Marchiblatt & Cantos (2003) | 0.1 | 0.021 | 5.3% | 0.10 [0.06, 0.14] | 2003 |
| Rodrigues Bachur (2008) | 0.268 | 0.018 | 5.7% | 0.27 [0.23, 0.30] | 2008 |
| Cardoso et al (2011) | 0.002 | 0.002 | 6.8% | 0.00 [-0.00, 0.01] | 2011 |
| **Subtotal (95% CI)** | | | **30.4%** | **0.09 [0.02, 0.16]** | |
| Heterogeneity: Tau² = 0.01; Chi² = 254.56, df = 4 (P < 0.00001); I² = 98% | | | | | |
| Test for overall effect: Z = 2.43 (P = 0.01) | | | | | |
| **Total (95% CI)** | | | **100.0%** | **0.06 [0.04, 0.09]** | |
| Heterogeneity: Tau² = 0.00; Chi² = 524.14, df = 15 (P < 0.00001); I² = 97% | | | | | |
| Test for overall effect: Z = 6.31 (P < 0.00001) | | | | | |
| Test for subgroup differences: Chi² = 0.64, df = 3 (P = 0.89), I² = 0% | | | | | |

Proportion
IV, Random, 95% CI

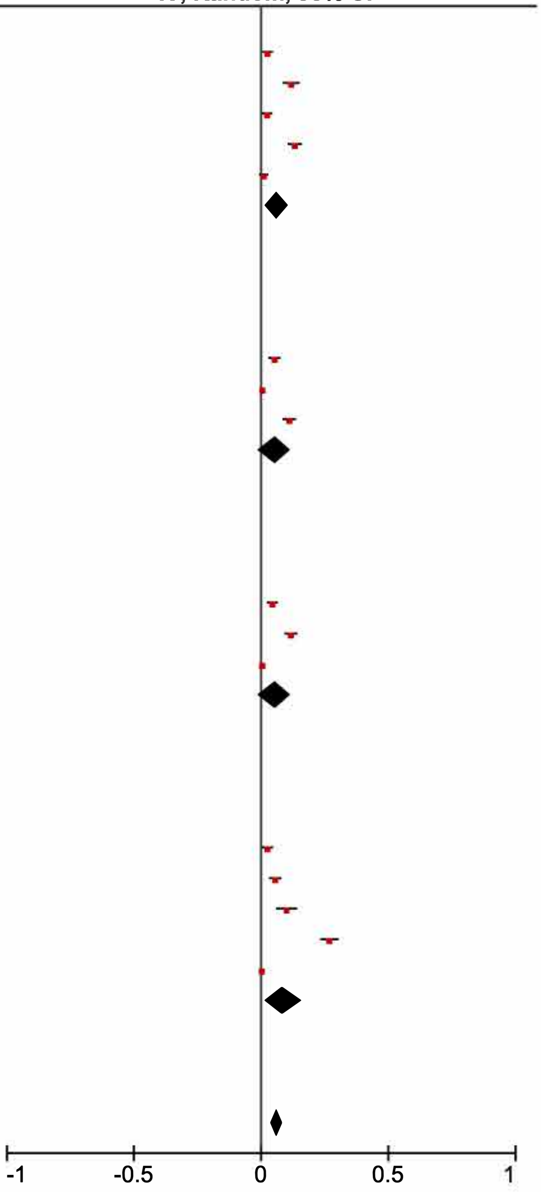


Supp. Figure 16

| Study or Subgroup | Proportion | SE | Weight | Proportion IV, Random, 95% CI |
|---|---|---|---|---|
| **Pooled prevalence of AL** | | | | |
| Chacin-Bonilla et al (1992) | 0.069 | 0.047 | 13.5% | 0.07 [-0.02, 0.16] |
| **Subtotal (95% CI)** | | | **13.5%** | **0.07 [-0.02, 0.16]** |
| Heterogeneity: Not applicable | | | | |
| Test for overall effect: Z = 1.47 (P = 0.14) | | | | |
| **Pooled prevalence of TT** | | | | |
| Chacin-Bonilla et al (1992) | 0.034 | 0.034 | 21.7% | 0.03 [-0.03, 0.10] |
| **Subtotal (95% CI)** | | | **21.7%** | **0.03 [-0.03, 0.10]** |
| Heterogeneity: Not applicable | | | | |
| Test for overall effect: Z = 1.00 (P = 0.32) | | | | |
| **Pooled prevalence of SS** | | | | |
| Arenas-Pinto et al (2003) | 0.099 | 0.017 | 43.1% | 0.10 [0.07, 0.13] |
| Chacin-Bonilla et al (1992) | 0.034 | 0.034 | 21.7% | 0.03 [-0.03, 0.10] |
| **Subtotal (95% CI)** | | | **64.8%** | **0.07 [0.01, 0.14]** |
| Heterogeneity: Tau² = 0.00; Chi² = 2.92, df = 1 (P = 0.09); I² = 66% | | | | |
| Test for overall effect: Z = 2.30 (P = 0.02) | | | | |
| **Total (95% CI)** | | | **100.0%** | **0.07 [0.03, 0.10]** |
| Heterogeneity: Tau² = 0.00; Chi² = 4.90, df = 3 (P = 0.18); I² = 39% | | | | |
| Test for overall effect: Z = 3.43 (P = 0.0006) | | | | |
| Test for subgroup differences: Chi² = 0.78, df = 2 (P = 0.68), I² = 0% | | | | |

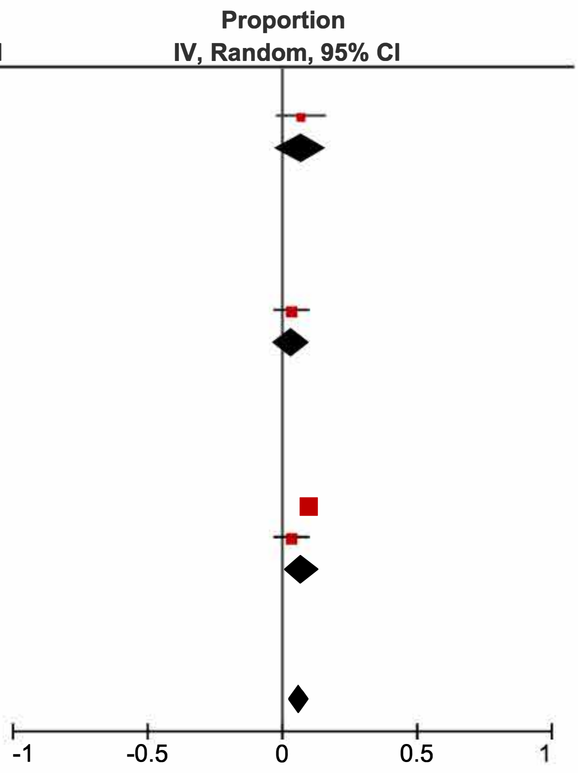


Supp. Figure 17

| Study or Subgroup | Proportion | SE | Weight | Proportion IV, Random, 95% CI |
|---|---|---|---|---|
| **Pooled prevalence of AL** | | | | |
| Paboriboune et al (2014) | 0.007 | 0.007 | 28.1% | 0.01 [-0.01, 0.02] |
| **Subtotal (95% CI)** | | | **28.1%** | **0.01 [-0.01, 0.02]** |
| Heterogeneity: Not applicable | | | | |
| Test for overall effect: Z = 1.00 (P = 0.32) | | | | |
| **Pooled prevalence of TT** | | | | |
| Paboriboune et al (2014) | 0.022 | 0.013 | 27.2% | 0.02 [-0.00, 0.05] |
| **Subtotal (95% CI)** | | | **27.2%** | **0.02 [-0.00, 0.05]** |
| Heterogeneity: Not applicable | | | | |
| Test for overall effect: Z = 1.69 (P = 0.09) | | | | |
| **Pooled prevalence of HW** | | | | |
| Paboriboune et al (2014) | 0.139 | 0.03 | 22.9% | 0.14 [0.08, 0.20] |
| **Subtotal (95% CI)** | | | **22.9%** | **0.14 [0.08, 0.20]** |
| Heterogeneity: Not applicable | | | | |
| Test for overall effect: Z = 4.63 (P < 0.00001) | | | | |
| **Pooled prevalence of SS** | | | | |
| Paboriboune et al (2014) | 0.204 | 0.034 | 21.7% | 0.20 [0.14, 0.27] |
| **Subtotal (95% CI)** | | | **21.7%** | **0.20 [0.14, 0.27]** |
| Heterogeneity: Not applicable | | | | |
| Test for overall effect: Z = 6.00 (P < 0.00001) | | | | |
| **Total (95% CI)** | | | **100.0%** | **0.08 [0.02, 0.15]** |

Heterogeneity: $Tau^2 = 0.00$; $Chi^2 = 48.44$, df = 3 ($P < 0.00001$); $I^2 = 94\%$
Test for overall effect: Z = 2.59 (P = 0.010)
Test for subgroup differences: $Chi^2 = 48.44$, df = 3 ($P < 0.00001$), $I^2 = 93.8\%$

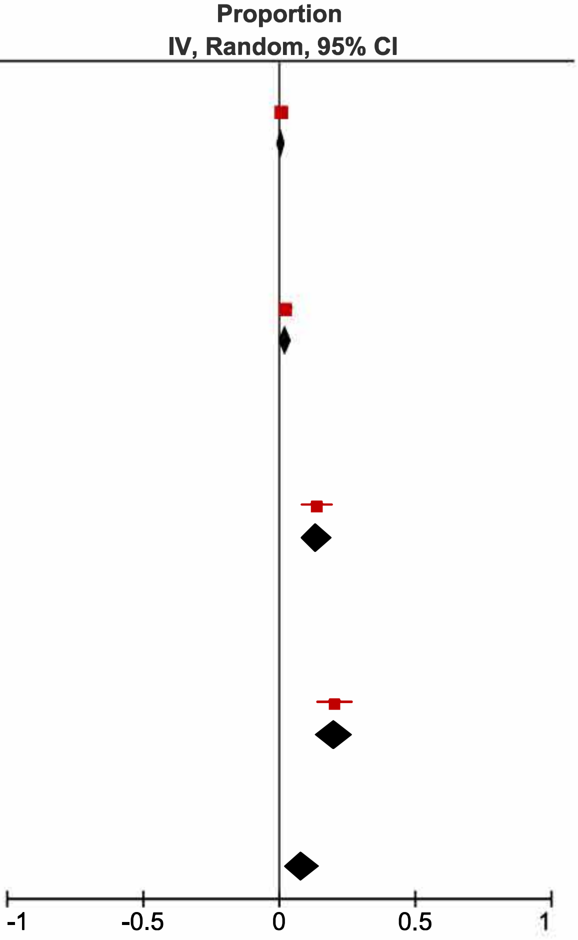


Supp. Figure 18

| Study or Subgroup | Proportion | SE | Weight | Proportion IV, Random, 95% CI |
|---|---|---|---|---|
| **Pooled prevalence of AL** | | | | |
| Wiwanitkit et al (2001) | 0.133 | 0.044 | 19.8% | 0.13 [0.05, 0.22] |
| **Subtotal (95% CI)** | | | **19.8%** | **0.13 [0.05, 0.22]** |
| Heterogeneity: Not applicable | | | | |
| Test for overall effect: Z = 3.02 (P = 0.003) | | | | |
| **Pooled prevalence of HW** | | | | |
| Wiwanitkit et al (2001) | 0.133 | 0.044 | 19.8% | 0.13 [0.05, 0.22] |
| **Subtotal (95% CI)** | | | **19.8%** | **0.13 [0.05, 0.22]** |
| Heterogeneity: Not applicable | | | | |
| Test for overall effect: Z = 3.02 (P = 0.003) | | | | |
| **Pooled prevalence of SS** | | | | |
| Manatsathit et al (1996) | 0.044 | 0.031 | 27.4% | 0.04 [-0.02, 0.10] |
| Wiwanitkit et al (2001) | 0.033 | 0.023 | 33.1% | 0.03 [-0.01, 0.08] |
| **Subtotal (95% CI)** | | | **60.5%** | **0.04 [0.00, 0.07]** |
| Heterogeneity: Tau² = 0.00; Chi² = 0.08, df = 1 (P = 0.78); I² = 0% | | | | |
| Test for overall effect: Z = 2.00 (P = 0.05) | | | | |
| **Total (95% CI)** | | | **100.0%** | **0.08 [0.02, 0.13]** |
| Heterogeneity: Tau² = 0.00; Chi² = 7.13, df = 3 (P = 0.07); I² = 58% | | | | |
| Test for overall effect: Z = 2.87 (P = 0.004) | | | | |
| Test for subgroup differences: Chi² = 7.05, df = 2 (P = 0.03), I² = 71.6% | | | | |

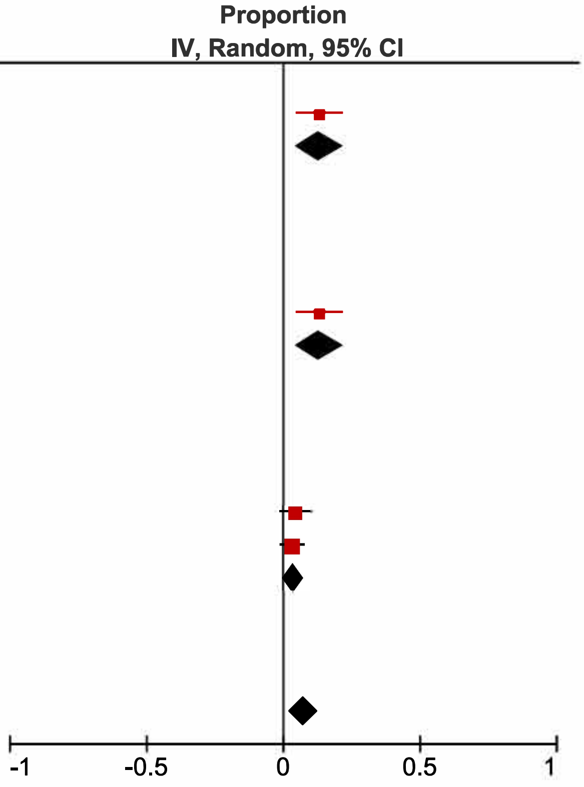


Supp. Figure 19

| Study or Subgroup | Proportion | SE | Weight | Proportion IV, Random, 95% CI |
|---|---|---|---|---|
| **Pooled prevalence of AL** | | | | |
| Asma et al (2011) | 0.139 | 0.019 | 31.9% | 0.14 [0.10, 0.18] |
| **Subtotal (95% CI)** | | | **31.9%** | **0.14 [0.10, 0.18]** |
| Heterogeneity: Not applicable | | | | |
| Test for overall effect: Z = 7.32 (P < 0.00001) | | | | |
| **Pooled prevalence of TT** | | | | |
| Asma et al (2011) | 0.064 | 0.013 | 33.4% | 0.06 [0.04, 0.09] |
| **Subtotal (95% CI)** | | | **33.4%** | **0.06 [0.04, 0.09]** |
| Heterogeneity: Not applicable | | | | |
| Test for overall effect: Z = 4.92 (P < 0.00001) | | | | |
| **Pooled prevalence of HW** | | | | |
| Asma et al (2011) | 0.006 | 0.004 | 34.7% | 0.01 [-0.00, 0.01] |
| **Subtotal (95% CI)** | | | **34.7%** | **0.01 [-0.00, 0.01]** |
| Heterogeneity: Not applicable | | | | |
| Test for overall effect: Z = 1.50 (P = 0.13) | | | | |
| **Total (95% CI)** | | | **100.0%** | **0.07 [-0.00, 0.14]** |

Heterogeneity: $Tau^2 = 0.00$; $Chi^2 = 61.79$, df = 2 ($P < 0.00001$); $I^2 = 97\%$
Test for overall effect: Z = 1.86 (P = 0.06)
Test for subgroup differences: $Chi^2 = 61.79$, df = 2 ($P < 0.00001$), $I^2 = 96.8\%$

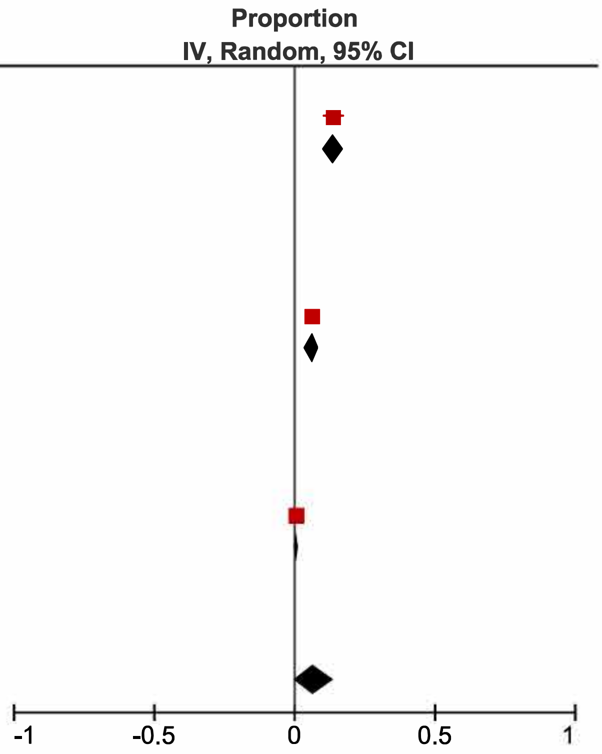


Supp. Figure 20

| Study or Subgroup | Proportion | SE | Weight | Proportion IV, Random, 95% CI |
|---|---|---|---|---|
| **Pooled prevalence of AL** | | | | |
| Dwivedi et al (2007) | 0.4 | 0.057 | 20.7% | 0.40 [0.29, 0.51] |
| **Subtotal (95% CI)** | | | **20.7%** | **0.40 [0.29, 0.51]** |
| Heterogeneity: Not applicable | | | | |
| Test for overall effect: Z = 7.02 (P < 0.00001) | | | | |
| **Pooled prevalence of HW** | | | | |
| Dwivedi et al (2007) | 0.187 | 0.045 | 23.1% | 0.19 [0.10, 0.28] |
| Janagond et al (2013) | 0.02 | 0.014 | 28.1% | 0.02 [-0.01, 0.05] |
| **Subtotal (95% CI)** | | | **51.2%** | **0.10 [-0.07, 0.26]** |
| Heterogeneity: Tau² = 0.01; Chi² = 12.56, df = 1 (P = 0.0004); I² = 92% | | | | |
| Test for overall effect: Z = 1.18 (P = 0.24) | | | | |
| **Pooled prevalence of SS** | | | | |
| Janagond et al (2013) | 0.02 | 0.014 | 28.1% | 0.02 [-0.01, 0.05] |
| **Subtotal (95% CI)** | | | **28.1%** | **0.02 [-0.01, 0.05]** |
| Heterogeneity: Not applicable | | | | |
| Test for overall effect: Z = 1.43 (P = 0.15) | | | | |
| **Total (95% CI)** | | | **100.0%** | **0.14 [0.04, 0.23]** |
| Heterogeneity: Tau² = 0.01; Chi² = 54.60, df = 3 (P < 0.00001); I² = 95% | | | | |
| Test for overall effect: Z = 2.81 (P = 0.005) | | | | |
| Test for subgroup differences: Chi² = 42.36, df = 2 (P < 0.00001), I² = 95.3% | | | | |

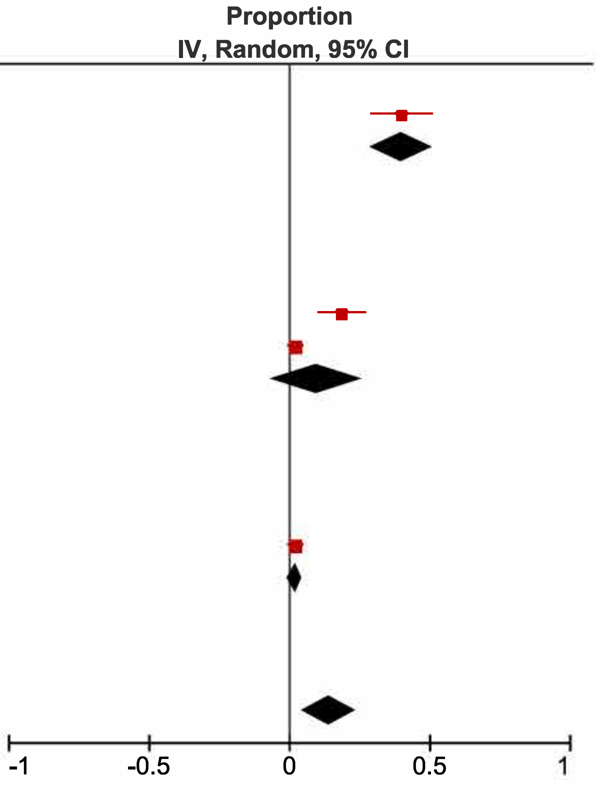


Supp. Figure 21

| Study or Subgroup | Proportion | SE | Weight | Proportion IV, Random, 95% CI |
|---|---|---|---|---|
| **Pooled prevalence of TT** | | | | |
| Tian et al (2012) | 0.007 | 0.005 | 55.7% | 0.01 [-0.00, 0.02] |
| **Subtotal (95% CI)** | | | **55.7%** | **0.01 [-0.00, 0.02]** |
| Heterogeneity: Not applicable | | | | |
| Test for overall effect: Z = 1.40 (P = 0.16) | | | | |
| **Pooled prevalence of HW** | | | | |
| Tian et al (2012) | 0.036 | 0.011 | 44.3% | 0.04 [0.01, 0.06] |
| **Subtotal (95% CI)** | | | **44.3%** | **0.04 [0.01, 0.06]** |
| Heterogeneity: Not applicable | | | | |
| Test for overall effect: Z = 3.27 (P = 0.001) | | | | |
| **Total (95% CI)** | | | **100.0%** | **0.02 [-0.01, 0.05]** |

Heterogeneity: $Tau^2 = 0.00$; $Chi^2 = 5.76$, df = 1 (P = 0.02); $I^2 = 83\%$
Test for overall effect: Z = 1.38 (P = 0.17)
Test for subgroup differences: $Chi^2 = 5.76$, df = 1 (P = 0.02), $I^2 = 82.6\%$

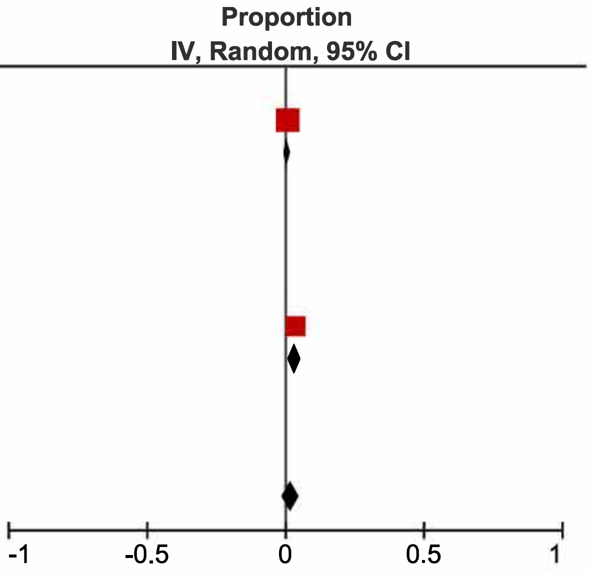


Supp. Figure 22

| Study or Subgroup | Proportion | SE | Weight | Proportion IV, Random, 95% CI |
|---|---|---|---|---|
| **Pooled prevalence of AL** | | | | |
| Tiwari et al (2013) | 0.027 | 0.006 | 24.9% | 0.03 [0.02, 0.04] |
| **Subtotal (95% CI)** | | | **24.9%** | **0.03 [0.02, 0.04]** |
| Heterogeneity: Not applicable | | | | |
| Test for overall effect: Z = 4.50 (P < 0.00001) | | | | |
| **Pooled prevalence of TT** | | | | |
| Tiwari et al (2013) | 0.047 | 0.008 | 23.3% | 0.05 [0.03, 0.06] |
| **Subtotal (95% CI)** | | | **23.3%** | **0.05 [0.03, 0.06]** |
| Heterogeneity: Not applicable | | | | |
| Test for overall effect: Z = 5.88 (P < 0.00001) | | | | |
| **Pooled prevalence of HW** | | | | |
| Tiwari et al (2013) | 0.026 | 0.006 | 24.9% | 0.03 [0.01, 0.04] |
| **Subtotal (95% CI)** | | | **24.9%** | **0.03 [0.01, 0.04]** |
| Heterogeneity: Not applicable | | | | |
| Test for overall effect: Z = 4.33 (P < 0.0001) | | | | |
| **Pooled prevalence of SS** | | | | |
| Tiwari et al (2013) | 0.003 | 0.002 | 27.0% | 0.00 [-0.00, 0.01] |
| **Subtotal (95% CI)** | | | **27.0%** | **0.00 [-0.00, 0.01]** |
| Heterogeneity: Not applicable | | | | |
| Test for overall effect: Z = 1.50 (P = 0.13) | | | | |
| **Total (95% CI)** | | | **100.0%** | **0.02 [0.01, 0.04]** |

Heterogeneity: Tau² = 0.00; Chi² = 48.58, df = 3 (P < 0.00001); I² = 94%
Test for overall effect: Z = 2.46 (P = 0.01)
Test for subgroup differences: Chi² = 48.58, df = 3 (P < 0.00001), I² = 93.8%

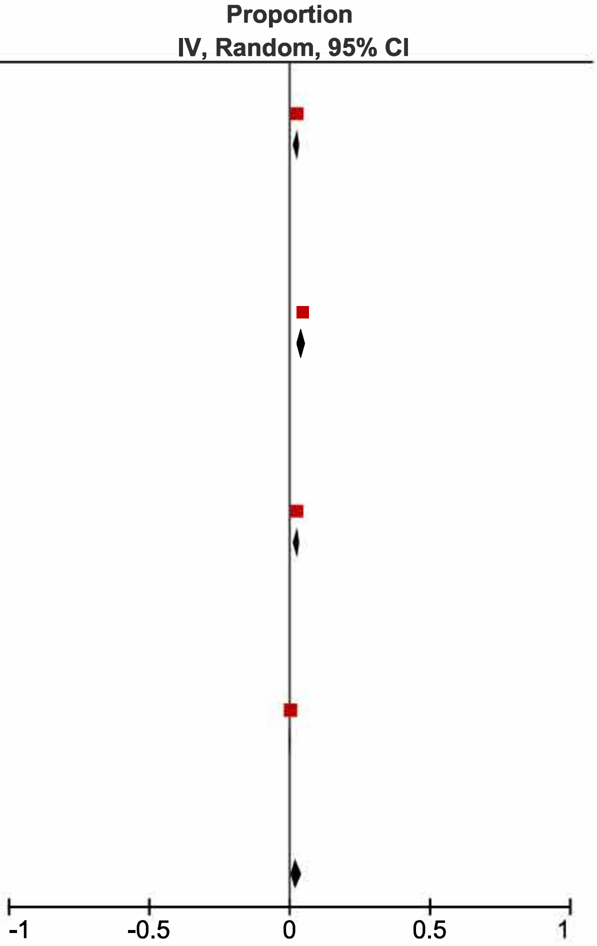


Supp. Figure 23

| Study or Subgroup | Proportion | SE | Weight | Proportion IV, Random, 95% CI |
|---|---|---|---|---|
| **Pooled prevalence of SS** | | | | |
| Zali et al (2004) | 0.01 | 0.007 | 100.0% | 0.01 [-0.00, 0.02] |
| **Subtotal (95% CI)** | | | **100.0%** | **0.01 [-0.00, 0.02]** |
| Heterogeneity: Not applicable | | | | |
| Test for overall effect: Z = 1.43 (P = 0.15) | | | | |
| **Total (95% CI)** | | | **100.0%** | **0.01 [-0.00, 0.02]** |
| Heterogeneity: Not applicable | | | | |
| Test for overall effect: Z = 1.43 (P = 0.15) | | | | |
| Test for subgroup differences: Not applicable | | | | |

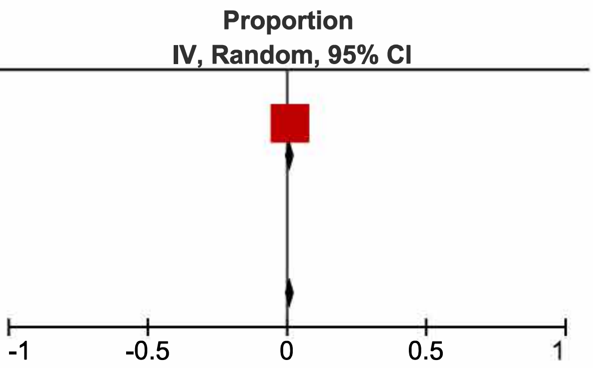


Supp. Figure 24

| Study or Subgroup | Proportion | SE | Weight | Proportion IV, Random, 95% CI |
|---|---|---|---|---|
| **Pooled prevalence of SS** | | | | |
| Sadlier et al (2013) | 0.022 | 0.016 | 100.0% | 0.02 [-0.01, 0.05] |
| **Subtotal (95% CI)** | | | **100.0%** | **0.02 [-0.01, 0.05]** |
| Heterogeneity: Not applicable | | | | |
| Test for overall effect: Z = 1.38 (P = 0.17) | | | | |
| | | | | |
| **Total (95% CI)** | | | **100.0%** | **0.02 [-0.01, 0.05]** |
| Heterogeneity: Not applicable | | | | |
| Test for overall effect: Z = 1.38 (P = 0.17) | | | | |
| Test for subgroup differences: Not applicable | | | | |

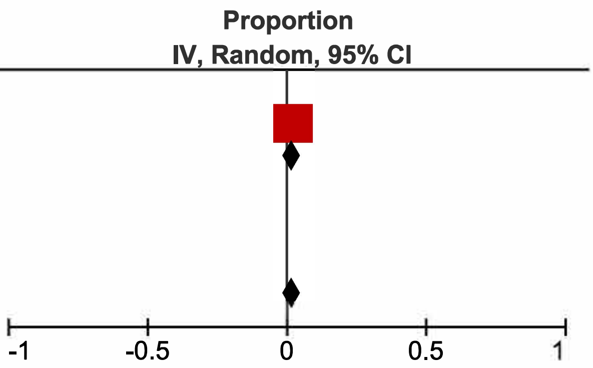


Supp. Figure 25

| Study or Subgroup | Proportion | SE | Weight | Proportion IV, Random, 95% CI |
|---|---|---|---|---|
| **Pooled prevalence of SS** | | | | |
| Nabha et al (2012) | 0.252 | 0.043 | 100.0% | 0.25 [0.17, 0.34] |
| **Subtotal (95% CI)** | | | **100.0%** | **0.25 [0.17, 0.34]** |
| Heterogeneity: Not applicable | | | | |
| Test for overall effect: Z = 5.86 (P < 0.00001) | | | | |
| **Total (95% CI)** | | | **100.0%** | **0.25 [0.17, 0.34]** |
| Heterogeneity: Not applicable | | | | |
| Test for overall effect: Z = 5.86 (P < 0.00001) | | | | |
| Test for subgroup differences: Not applicable | | | | |

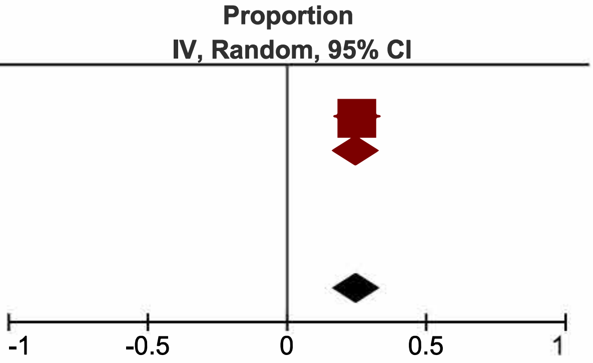


Supp. Figure 26

| Country | AL | TT | HW | SS | Total |
|---|---|---|---|---|---|
| Nigeria | 0.06 (0.03, 0.09) | 0.02 (0.01, 0.03) | 0.04 (0.02, 0.06) | 0.02 (0.00, 0.03) | 0.03 (0.02, 0.04) |
| Ethiopia | 0.09 (0.06, 0.12) | 0.03 (0.02, 0.05) | 0.02 (0.01, 0.03) | 0.08 (0.05, 0.11) | 0.06 (0.05, 0.07) |
| Mozambique | 0.04 (0.02, 0.06) | 0.11 (0.08, 0.14) | 0.00 (-0.00, 0.01) | 0.01 (0.00, 0.02) | 0.04 (0.01, 0.02) |
| South Africa | 0.1 (0.06, 0.14) | 0.01 (-0.00, 0.03) | 0.01 (-0.00. 0.03) | 0.01 (-0.00, 0.03) | 0.03 (-0.00, 0.03) |
| Malawi | 0.03 (-0.11, 0.17) |  | 0.14 (0.06, 0.23) | 0.03 (-0.02, 0.08) | 0.08 (0.02, 0.14) |
| Tanzania | 0.13 (0.10, 0.16) |  | 0.15 (0.12, 0.19) | 0.08 (-0.02, 0.19) | 0.12 (0.07, 0.16) |
| Guinea-bissau |  |  | 0.11 (0.01, 0.21) | 0.14 (0.03, 0.24) | 0.12 (-.05, 0.19) |
| Guinea | 0.21 (0.17, 0.25) | 0.48 (0.42, 0.54) | 0.04 (0.02, 0.06) | 0.1 (0.07, 0.12) | 0.21 (0.11, 0.3) |
| Kenya | 0.04 (0.03, 0.04) | 0.02 (0.01, 0.02) | 0.04 (0.03, 0.05) | 0.0 (0, 0.01) | 0.01 (0.01, 0.01) |
| Zambia | 0.12 (0.09, 0.16) |  | 0.1 (0.06, 0.13) | 0.02 (0, 0.03) | 0.04 (0.03, 0.05) |
| Uganda |  | 0.01 (-0, 0.01) | 0.28 (0.22, 0.34) | 0.04 (0.01, 0.07) | 0.1 (0, 0.2) |
| Cameroon | 0.13 (0.08, 0.18) | 0.04 (0.01, 0.06) | 0.01 (-0, 0.03) | 0.03 (0.01, 0.05) | 0.04 (0.01, 0.07) |
| Republic of Congo | 0.04 (0.01, 0.07) | 0.01 (-0.01, 0.02) | 0.01 (-0, 0.03) |  | 0.01 (-0, 0.03) |
| Cuba | 0.03 (-0.01,0.07) |  |  |  | 0.03 (-0.01, 0.07) |
| Brazil | 0.06 (0.02, 0.11) | 0.05 (-0.01, 0.12) | 0.05 (-0.01, 0.12) | 0.09 (0.02, 0.16) | 0.06 (0.04, 0.09) |
| Venezuela | 0.07 (-0.02, 0.16) | 0.03 (-0.03, 0.1) |  | 0.07 (0.01, 0.14) | 0.07 (0.03, 0.10) |
| Laos | 0.01 (-0.01, 0.02) | 0.02 (-0.00, 0.05) | 0.14 (0.08, 0.20) | 0.2 (0.14, 0.27) | 0.08 (0.02, 0.15) |
| Thailand | 0.13 (0.05, 0.22) |  | 0.13 (0.05, 0.22) | 0.04 (0.0, 0.07) | 0.08 (0.02, 0.13) |
| Malaysia | 0.14 (0.10, 0.18) | 0.06 (0.04, 0.09) | 0.01 (-0, 0.01) |  | 0.07 (-0, 0.14) |
| India | 0.40 (0.29, 0.51) |  | 0.10 (-0.07, 0.26) | 0.02 (-0.1, 0.05) | 0.14 (0.04, 0.23) |
| China |  | 0.01 (-0, 0.02) | 0.04 (0.01, 0.06) |  | 0.02 (-0.01, 0.05) |
| Nepal | 0.03 (0.02, 0.04) | 0.05 (0.03, 0.06) | 0.03 (0.01, 0.04) | 0.0 (-0.00, 0.01) | 0.02 (0.01, 0.04) |
| Iran |  |  |  | 0.01 (-0, 0.02) | 0.01 (-0, 0.02) |
| Ireland |  |  |  | 0.02 (-0.01, 0.05) | 0.02 (-0.01, 0.05) |
| USA |  |  |  | 0.25 (0.17, 0.34) | 0.25 (0.17, 0.34) |

Supp. Table 2. Country-wise calculated pooled prevalence

| S. No. | Author | Was the sample frame appropriate to address the target population? | Were study participants sampled in an appropriate way? | Was the sample size adequate? | Were the study subjects and the setting described in detail? | Was the data analysis conducted with sufficient coverage of the identified sample? | Were valid methods used for the identification of the condition? | Was the condition measured in a standard, reliable way for all participants? | Was there appropriate statistical analysis? | Was the response rate adequate, and if not, was the low response rate managed appropriately? | Total |
|---|---|---|---|---|---|---|---|---|---|---|---|
| 1. | Abaver *et al* | 1 | 1 | 0 | 1 | 0 | 1 | 1 | 1 | 1 | 7 |
| 2. | Adamu *et al* | 1 | 0 | 0 | 1 | 1 | 1 | 1 | 1 | 1 | 7 |
| 3. | Adeleke *et al* | 1 | 1 | 1 | 0 | 1 | 1 | 1 | 1 | 1 | 8 |
| 4. | Akinbo *et al* | 1 | 0 | 1 | 0 | 1 | 1 | 1 | 1 | 1 | 7 |
| 5. | Alemayehu *et al* | 1 | 1 | 1 | 1 | 1 | 1 | 1 | 1 | 1 | 9 |
| 6. | Amancio *et al* | 1 | 0 | 1 | 0 | 1 | 1 | 1 | 1 | 1 | 7 |
| 7. | Amoo *et al* | 1 | 1 | 1 | 1 | 1 | 1 | 1 | 1 | 1 | 9 |
| 8. | Arenas- Pinto *et al* | 1 | 1 | 1 | 0 | 1 | 1 | 1 | 1 | 1 | 8 |
| 9. | Arndt *et al* | 1 | 1 | 1 | 1 | 1 | 1 | 1 | 1 | 1 | 9 |
| 10. | Asma *et al* | 1 | 1 | 1 | 0 | 1 | 1 | 1 | 1 | 1 | 8 |
| 11. | Assefa *et al* | 1 | 1 | 1 | 1 | 1 | 1 | 1 | 1 | 1 | 9 |
| 12. | Babatunde *et al* | 1 | 1 | 0 | 1 | 1 | 1 | 1 | 1 | 1 | 8 |
| 13. | Cardoso *et al* | 1 | 1 | 1 | 0 | 1 | 1 | 1 | 1 | 1 | 8 |

| | | | | | | | | | | | |
|---|---|---|---|---|---|---|---|---|---|---|---|
| 14. | Carveja *et al* | 1 | 1 | 1 | 0 | 1 | 1 | 1 | 1 | 1 | 8 |
| 15. | Chachin- Bonilla | 1 | 1 | 0 | 1 | 1 | 1 | 1 | 1 | 1 | 8 |
| 16. | Chintu *et al* | 1 | 1 | 0 | 1 | 0 | 1 | 1 | 1 | 1 | 7 |
| 17. | Cimerman *et al* | 1 | 0 | 1 | 0 | 1 | 1 | 1 | 1 | 1 | 7 |
| 18. | Da Silva *et al* | 0 | 0 | 1 | 0 | 1 | 1 | 1 | 1 | 1 | 6 |
| 19. | Dwivedi *et al* | 1 | 1 | 0 | 1 | 1 | 1 | 1 | 1 | 1 | 8 |
| 20. | Dowling *el al* | 1 | 1 | 1 | 1 | 1 | 1 | 1 | 1 | 1 | 9 |
| 21. | Escobado *et al* | 1 | 1 | 0 | 1 | 1 | 1 | 1 | 1 | 1 | 8 |
| 22. | Eshetu *et al* | 1 | 1 | 1 | 0 | 1 | 1 | 1 | 1 | 1 | 8 |
| 23. | Feitosa *et al* | 1 | 0 | 1 | 0 | 1 | 1 | 1 | 1 | 1 | 7 |
| 24. | Fekadu *et al* | 1 | 1 | 1 | 1 | 1 | 1 | 1 | 1 | 1 | 9 |
| 25. | Gedle *et al* | 1 | 1 | 1 | 0 | 1 | 1 | 1 | 1 | 1 | 8 |
| 26. | Getaneh *et al* | 1 | 1 | 1 | 1 | 1 | 1 | 1 | 1 | 1 | 9 |
| 27. | Ghorpade *et al* | 1 | 0 | 0 | 0 | 0 | 1 | 1 | 0 | 1 | 4 |
| 28. | Hailegebriel *et al* | 1 | 1 | 0 | 0 | 1 | 1 | 1 | 1 | 1 | 7 |
| 29. | Hailemariam *et al* | 1 | 1 | 0 | 1 | 1 | 1 | 1 | 1 | 1 | 8 |
| 30. | Hosseinipour *et al* | 1 | 0 | 1 | 0 | 1 | 1 | 1 | 1 | 1 | 7 |
| 31. | Hunter *et al* | 1 | 0 | 0 | 0 | 1 | 1 | 1 | 1 | 1 | 6 |

| | | | | | | | | | | | |
|---|---|---|---|---|---|---|---|---|---|---|---|
| 32. | Idindili *et al* | 1 | 0 | 1 | 0 | 1 | 1 | 1 | 1 | 1 | 7 |
| 33. | Janagond *et al* | 1 | 0 | 1 | 0 | 1 | 1 | 1 | 1 | 1 | 7 |
| 34. | Keshniro *et al* | 1 | 1 | 0 | 0 | 0 | 1 | 1 | 1 | 1 | 6 |
| 35. | Kipyegen *et al* | 1 | 1 | 1 | 0 | 1 | 1 | 1 | 1 | 1 | 8 |
| 36. | Lebbaad *et al* | 1 | 1 | 0 | 0 | 1 | 1 | 1 | 1 | 1 | 7 |
| 37. | Marchi Blatt & Cantos | 1 | 1 | 1 | 0 | 1 | 1 | 1 | 1 | 1 | 8 |
| 38. | Manatsathit *et al* | 1 | 1 | 0 | 0 | 1 | 1 | 1 | 1 | 1 | 7 |
| 39. | Mariam *et al* | 1 | 1 | 1 | 0 | 1 | 1 | 1 | 1 | 1 | 8 |
| 40. | Meamar *et al* | 1 | 0 | 0 | 0 | 1 | 1 | 1 | 0 | 1 | 5 |
| 41. | Mehta *et al* | 1 | 0 | 1 | 0 | 1 | 1 | 1 | 0 | 1 | 6 |
| 42. | Mengist *et al* | 1 | 1 | 1 | 0 | 1 | 1 | 1 | 1 | 1 | 8 |
| 43. | Modjarrad *et al* | 1 | 1 | 1 | 1 | 1 | 1 | 1 | 1 | 1 | 9 |
| 44. | Moges *et al* | 1 | 1 | 0 | 1 | 1 | 1 | 1 | 1 | 1 | 8 |
| 45. | Mohandas *et al* | 1 | 0 | 1 | 0 | 1 | 1 | 1 | 0 | 1 | 6 |
| 46. | Morawski *et al* | 1 | 1 | 1 | 1 | 1 | 1 | 1 | 1 | 1 | 9 |
| 47. | Moura *et al* | 1 | 0 | 0 | 0 | 1 | 1 | 1 | 0 | 1 | 5 |
| 48. | Mwambete *et al* | 1 | 1 | 0 | 0 | 1 | 1 | 1 | 1 | 1 | 7 |
| 49. | Mwambete *et al* | 1 | 1 | 0 | 0 | 1 | 0 | 1 | 1 | 1 | 6 |

| | | | | | | | | | | | |
|---|---|---|---|---|---|---|---|---|---|---|---|
| 50. | Nabha *et al* | 1 | 1 | 1 | 0 | 1 | 1 | 1 | 1 | 1 | 8 |
| 51. | Nkenfou *et al* | 1 | 1 | 0 | 0 | 1 | 1 | 1 | 1 | 1 | 7 |
| 52. | Ojurongbe *et al* | 1 | 1 | 0 | 1 | 1 | 1 | 1 | 1 | 1 | 8 |
| 53. | Oyedeji *et al* | 1 | 1 | 0 | 0 | 1 | 1 | 1 | 1 | 1 | 7 |
| 54. | Paboriboune *et al* | 0 | 1 | 1 | 1 | 1 | 1 | 1 | 1 | 1 | 8 |
| 55. | de Paula *et al* | 1 | 0 | 0 | 0 | 1 | 1 | 1 | 1 | 1 | 6 |
| 56. | Pinlaor *et al* | 1 | 0 | 0 | 0 | 1 | 1 | 1 | 1 | 1 | 6 |
| 57. | Prasad *et al* | 1 | 0 | 0 | 0 | 1 | 1 | 1 | 1 | 1 | 6 |
| 58. | Rodrigues *et al* | 1 | 1 | 1 | 1 | 1 | 1 | 1 | 1 | 1 | 9 |
| 59. | Roka *et al* | 1 | 1 | 1 | 0 | 1 | 1 | 1 | 1 | 1 | 8 |
| 60. | Roka *et al* | 1 | 1 | 1 | 0 | 1 | 1 | 1 | 1 | 1 | 8 |
| 61. | Sadlier *et el* | 1 | 1 | 0 | 1 | 1 | 1 | 1 | 1 | 1 | 8 |
| 62. | Sanyaolu *et el* | 1 | 1 | 0 | 1 | 1 | 1 | 1 | 1 | 1 | 8 |
| 63. | Shah *et al* | 1 | 0 | 0 | 0 | 1 | 1 | 1 | 1 | 1 | 6 |
| 64. | Tadesse *et al* | 1 | 1 | 0 | 0 | 1 | 1 | 1 | 1 | 1 | 7 |
| 65. | Teklemariam *et al* | 1 | 0 | 1 | 1 | 1 | 1 | 1 | 1 | 1 | 8 |
| 66. | Tian *et al* | 1 | 1 | 0 | 1 | 1 | 1 | 1 | 1 | 1 | 8 |
| 67. | Tiwari *et al* | 1 | 1 | 1 | 1 | 1 | 1 | 1 | 1 | 1 | 9 |

| | | | | | | | | | | | |
|---|---|---|---|---|---|---|---|---|---|---|---|
| 68. | Udeh *et al* | 1 | 1 | 1 | 0 | 1 | 1 | 1 | 1 | 1 | 8 |
| 69. | Vouking *et al* | 1 | 1 | 1 | 0 | 1 | 1 | 1 | 1 | 1 | 8 |
| 70. | Walson *et al* | 1 | 0 | 1 | 0 | 1 | 1 | 1 | 1 | 1 | 7 |
| 71. | Wiwanitkit *et al* | 1 | 1 | 0 | 1 | 1 | 1 | 1 | 1 | 1 | 8 |
| 72. | Wumba *et al* | 0 | 1 | 1 | 1 | 1 | 1 | 1 | 1 | 1 | 8 |
| 73. | Zali *et al* | 1 | 1 | 1 | 0 | 1 | 1 | 1 | 1 | 1 | 8 |
| 74. | zeynudin *et al* | 1 | 1 | 0 | 0 | 1 | 1 | 1 | 1 | 1 | 7 |

Supp Table 3. JBI quality score

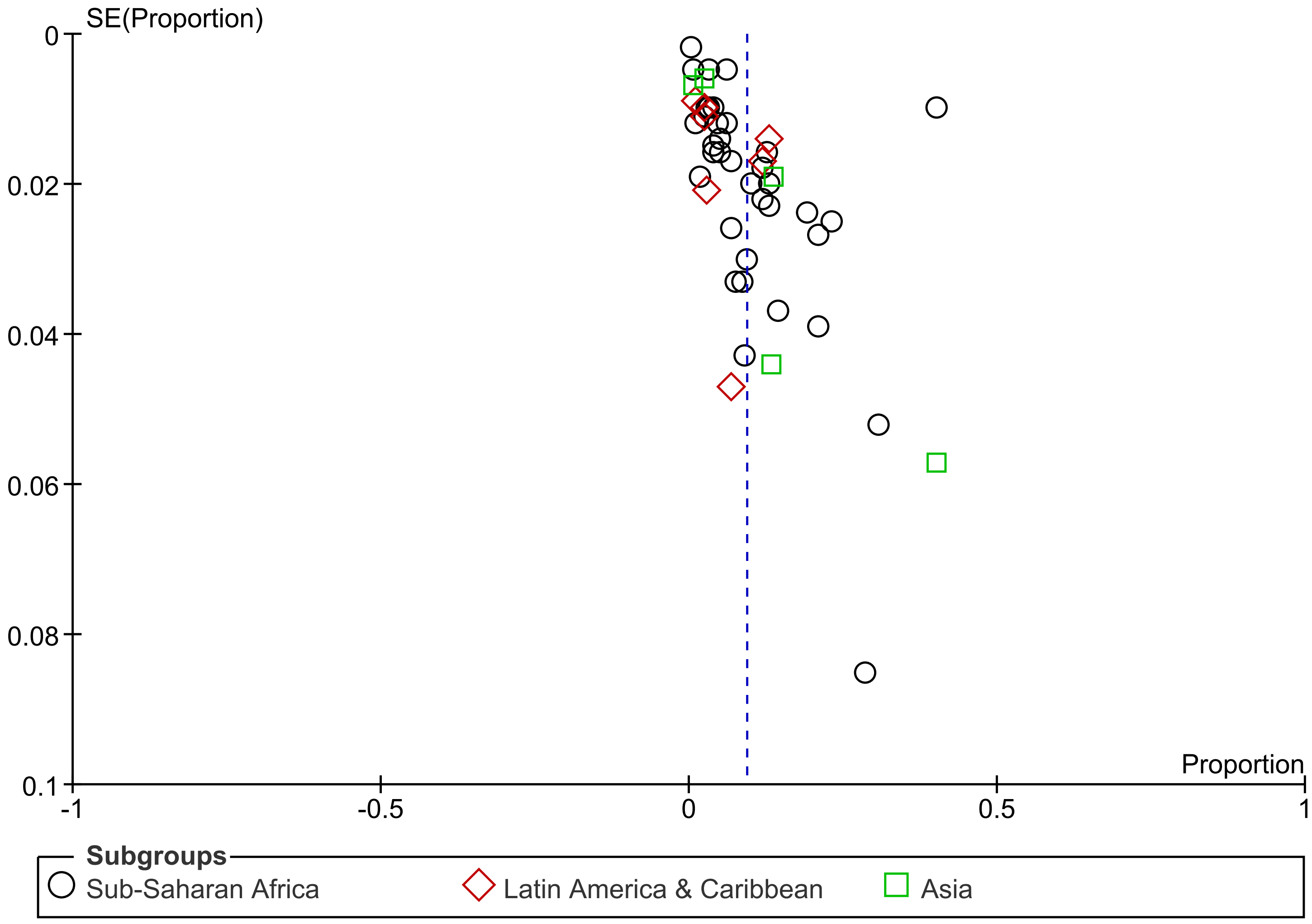


Supp. Figure 27

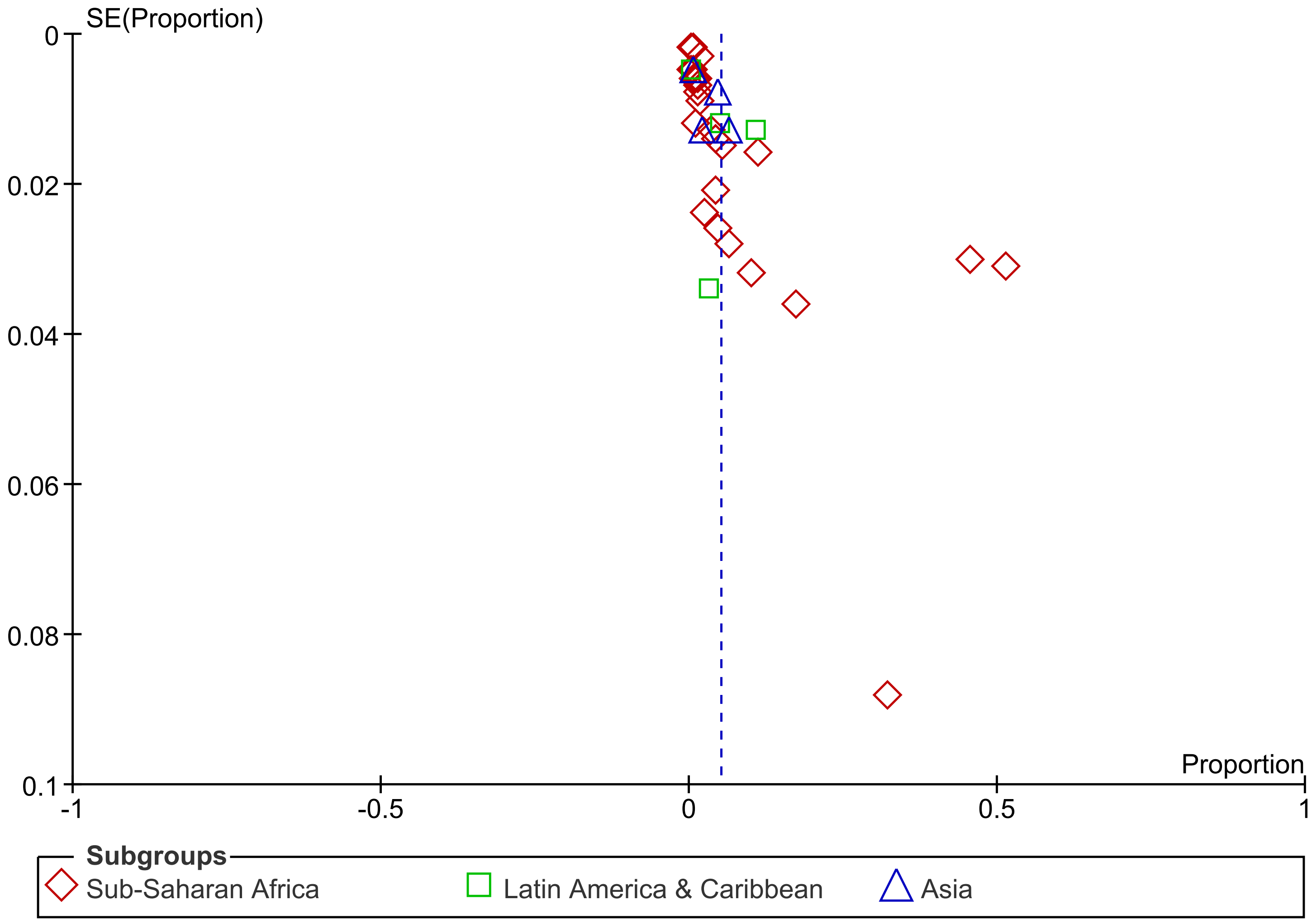


Supp. Figure 28

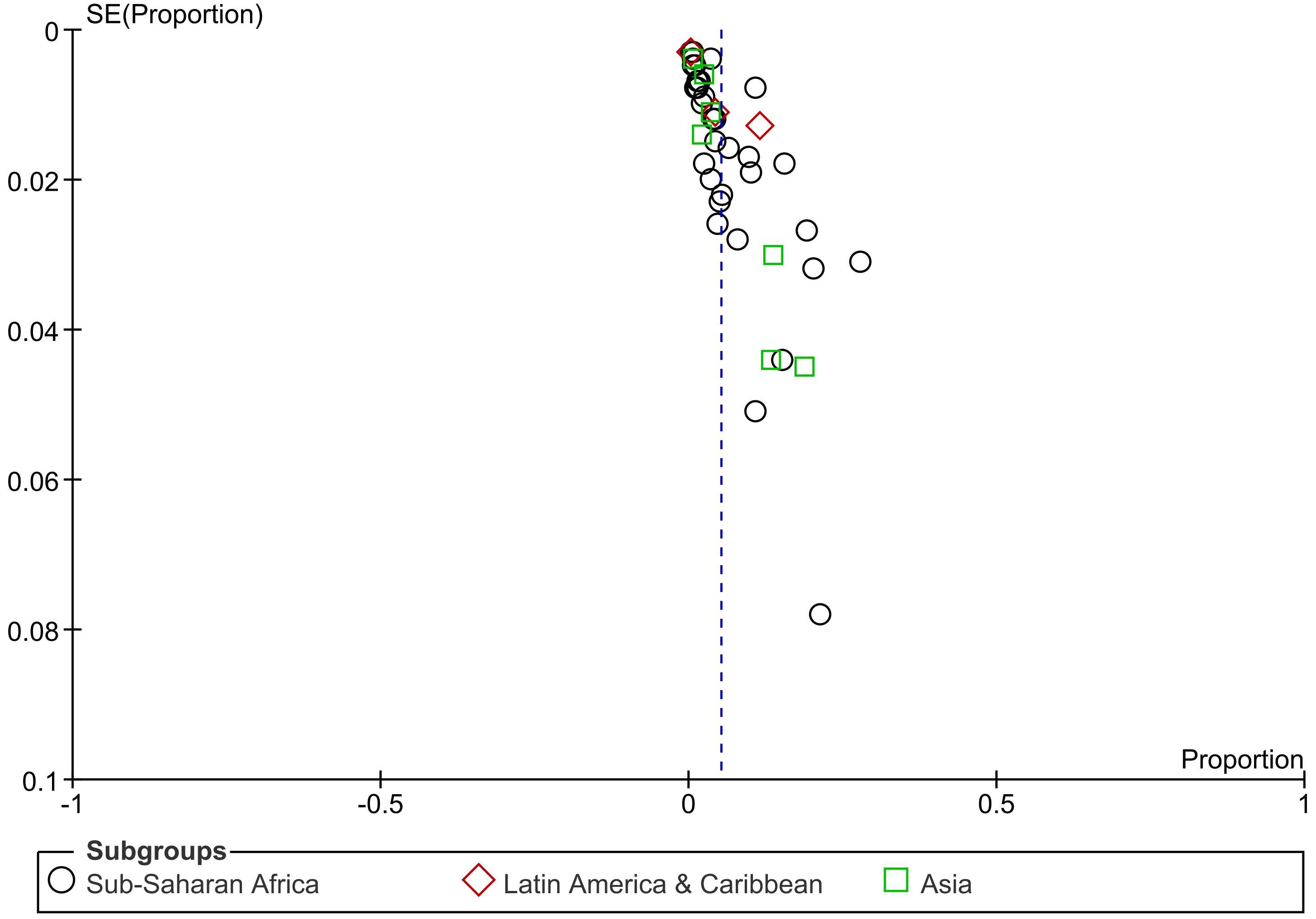


Supp. Figure 29

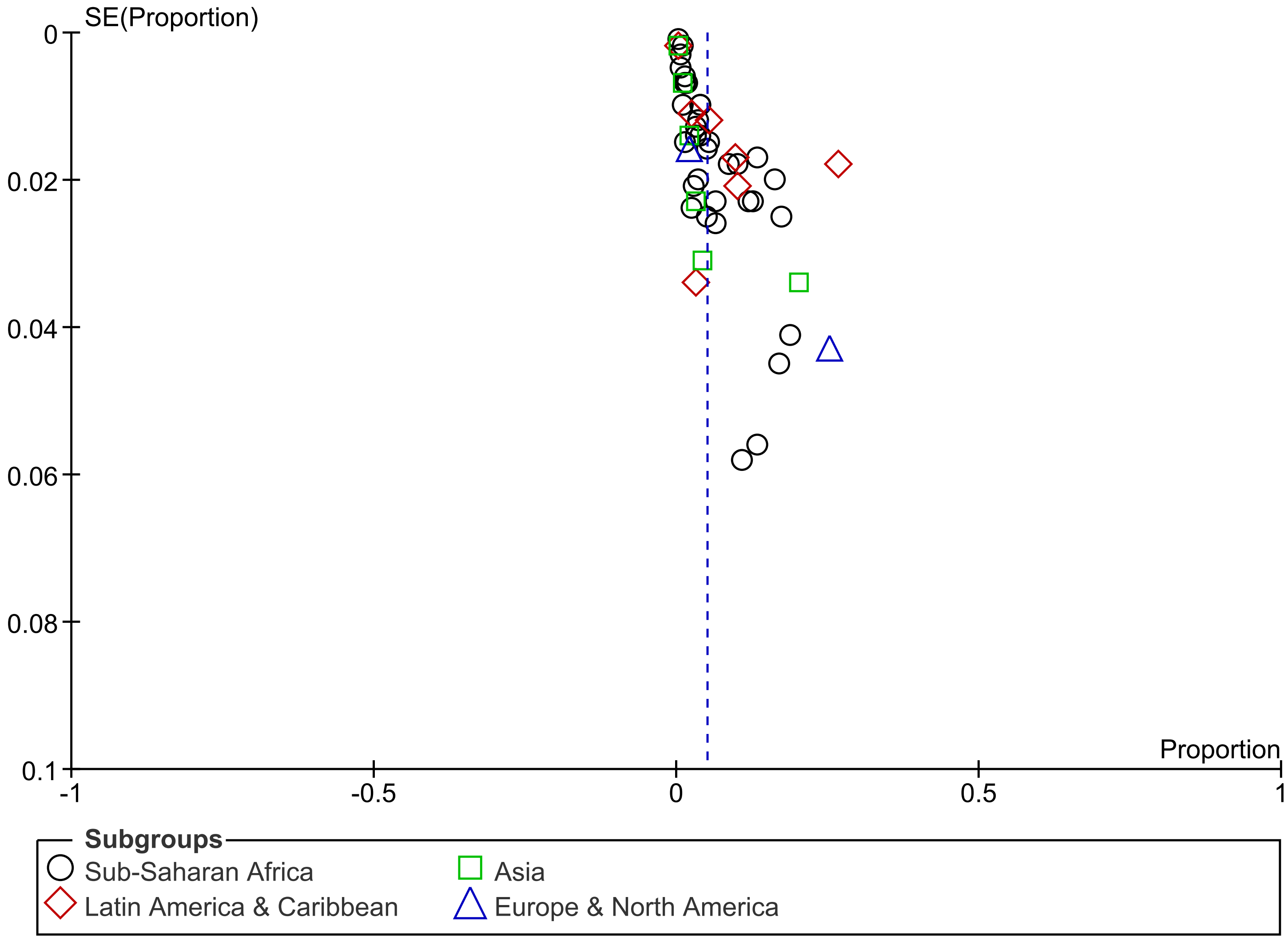


Supp. Figure 30

| Study or Subgroup | HIV positive AL positive Events | Total | HIV negative AL positive Events | Total | Weight | Odds Ratio M-H, Random, 95% CI |
|---|---|---|---|---|---|---|
| Abaver et al (2011) | 1 | 85 | 2 | 34 | 0.7% | 0.19 [0.02, 2.17] |
| Tian et al (2012) | 0 | 302 | 2 | 303 | 0.4% | 0.20 [0.01, 4.17] |
| Marchiblatt & Cantos (2003) | 5 | 211 | 11 | 213 | 3.2% | 0.45 [0.15, 1.31] |
| Cerveja et al (2017) | 15 | 371 | 12 | 146 | 5.7% | 0.47 [0.21, 1.03] |
| Roka et al (2012) | 50 | 260 | 14 | 50 | 7.1% | 0.61 [0.31, 1.22] |
| Hailegebriel et al (2017) | 11 | 216 | 10 | 125 | 4.6% | 0.62 [0.25, 1.50] |
| Roka et al (2013) | 63 | 273 | 19 | 60 | 8.6% | 0.65 [0.35, 1.19] |
| Moges et al (2006) | 8 | 28 | 141 | 376 | 5.0% | 0.67 [0.29, 1.55] |
| Udeh et al (2019) | 4 | 891 | 1 | 150 | 0.8% | 0.67 [0.07, 6.05] |
| Mariam et al (2008) | 23 | 109 | 14 | 51 | 5.9% | 0.71 [0.33, 1.52] |
| Sanyaolu et al (2011) | 5 | 65 | 97 | 1015 | 4.1% | 0.79 [0.31, 2.01] |
| Assefa et al (2009) | 26 | 214 | 21 | 164 | 8.6% | 0.94 [0.51, 1.74] |
| Babatunde et al (2010) | 6 | 90 | 6 | 90 | 2.7% | 1.00 [0.31, 3.23] |
| Chintu et al (1995) | 4 | 44 | 10 | 134 | 2.6% | 1.24 [0.37, 4.17] |
| Hoesseinipour et al (2007) | 8 | 266 | 3 | 123 | 2.1% | 1.24 [0.32, 4.76] |
| Feitosa et al (2001) | 43 | 365 | 495 | 5243 | 20.5% | 1.28 [0.92, 1.78] |
| Escobado & Nunez (1999) | 2 | 67 | 3 | 136 | 1.2% | 1.36 [0.22, 8.37] |
| Akinbo et al (2011) | 119 | 2000 | 21 | 500 | 12.8% | 1.44 [0.90, 2.32] |
| Hailemariam et al (2004) | 24 | 78 | 6 | 26 | 3.5% | 1.48 [0.53, 4.15] |
| **Total (95% CI)** | | **5935** | | **8939** | **100.0%** | **0.89 [0.73, 1.09]** |
| Total events | 417 | | 888 | | | |

Heterogeneity: Tau² = 0.02; Chi² = 20.41, df = 18 (P = 0.31); I² = 12%
Test for overall effect: Z = 1.13 (P = 0.26)

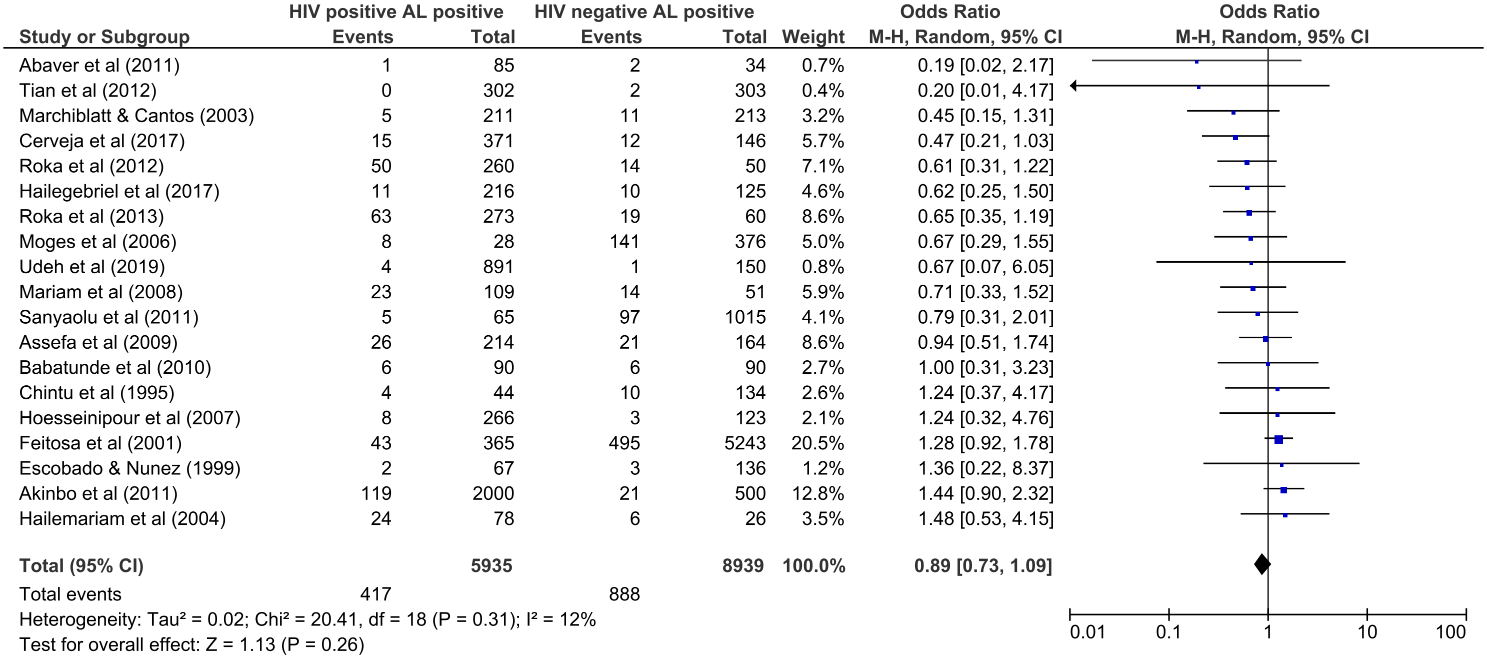


Supp. Figure 31

| | HIV positive TT positive | | HIV negative TT positive | | | Odds Ratio |
|---|---|---|---|---|---|---|
| Study or Subgroup | Events | Total | Events | Total | Weight | M-H, Random, 95% CI |
| Udeh et al (2019) | 3 | 891 | 6 | 150 | 5.1% | 0.08 [0.02, 0.33] |
| Marchiblatt & Cantos (2003) | 1 | 211 | 3 | 213 | 2.5% | 0.33 [0.03, 3.23] |
| Mwambete et al (2010) | 0 | 66 | 1 | 80 | 1.3% | 0.40 [0.02, 9.95] |
| Assefa et al (2009) | 8 | 214 | 13 | 164 | 8.2% | 0.45 [0.18, 1.12] |
| Cerveja et al (2017) | 41 | 371 | 26 | 146 | 11.5% | 0.57 [0.34, 0.98] |
| Hailemariam et al (2004) | 5 | 78 | 2 | 26 | 3.9% | 0.82 [0.15, 4.51] |
| Roka et al (2013) | 124 | 273 | 30 | 60 | 11.3% | 0.83 [0.48, 1.46] |
| Feitosa et al (2001) | 19 | 365 | 291 | 5243 | 12.0% | 0.93 [0.58, 1.51] |
| Sanyaolu et al (2011) | 3 | 65 | 40 | 1015 | 6.2% | 1.18 [0.35, 3.92] |
| Abaver et al (2011) | 1 | 85 | 0 | 34 | 1.3% | 1.22 [0.05, 30.81] |
| Roka et al (2012) | 134 | 260 | 21 | 50 | 10.8% | 1.47 [0.80, 2.71] |
| Babatunde et al (2010) | 9 | 90 | 6 | 90 | 6.9% | 1.56 [0.53, 4.57] |
| Moges et al (2006) | 9 | 28 | 76 | 376 | 8.8% | 1.87 [0.81, 4.30] |
| Tian et al (2012) | 2 | 302 | 1 | 303 | 2.2% | 2.01 [0.18, 22.32] |
| Hailegebriel et al (2017) | 4 | 226 | 0 | 125 | 1.6% | 5.08 [0.27, 95.06] |
| Mariam et al (2008) | 19 | 109 | 2 | 51 | 4.7% | 5.17 [1.16, 23.13] |
| Akinbo et al (2011) | 19 | 2000 | 0 | 500 | 1.7% | 9.85 [0.59, 163.43] |
| **Total (95% CI)** | | **5634** | | **8626** | **100.0%** | **0.96 [0.65, 1.43]** |
| Total events | 401 | | 518 | | | |

Heterogeneity: Tau² = 0.27; Chi² = 34.19, df = 16 (P = 0.005); I² = 53%
Test for overall effect: Z = 0.18 (P = 0.86)

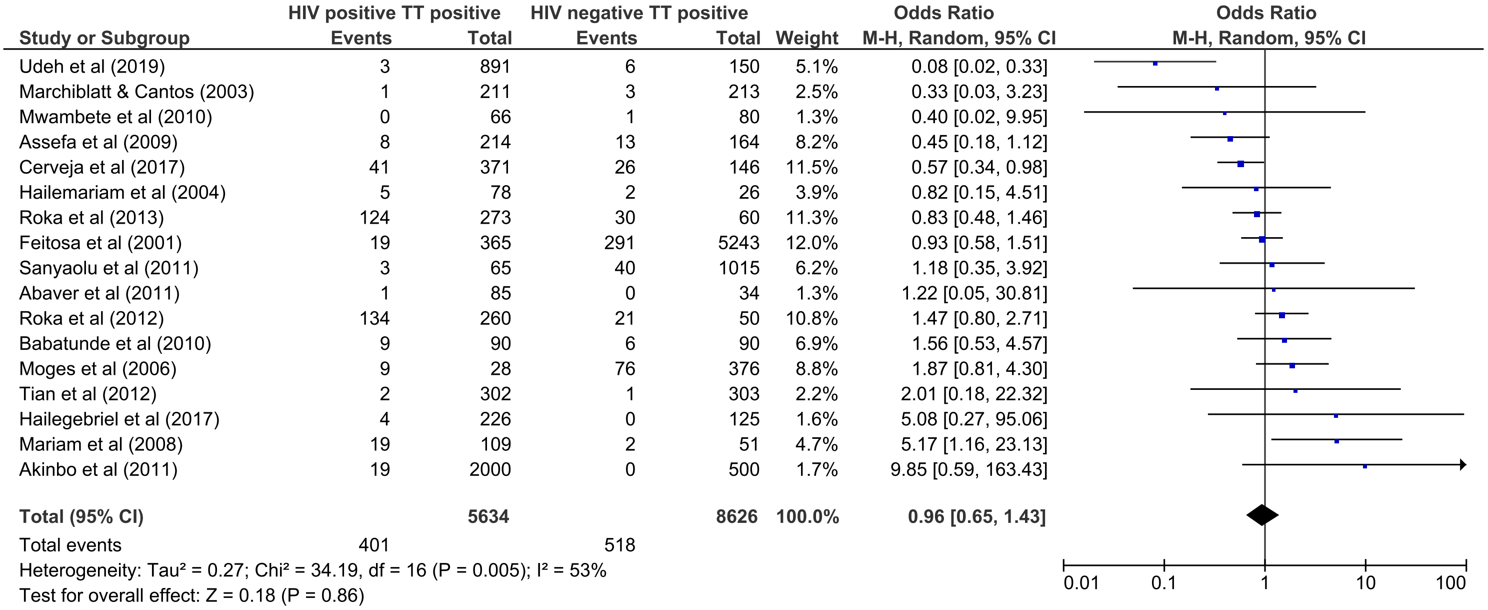


Supp. Figure 32

| Study or Subgroup | HIV positive HW positive Events | Total | HIV negative HW positive Events | Total | Weight | Odds Ratio M-H, Random, 95% CI |
|---|---|---|---|---|---|---|
| Udeh et al (2019) | 5 | 891 | 10 | 150 | 5.7% | 0.08 [0.03, 0.23] |
| Lebbad et al (2001) | 4 | 37 | 7 | 15 | 4.5% | 0.14 [0.03, 0.59] |
| Marchiblatt & Cantos (2003) | 0 | 211 | 3 | 213 | 1.7% | 0.14 [0.01, 2.77] |
| Cerveja et al (2017) | 1 | 371 | 2 | 146 | 2.4% | 0.19 [0.02, 2.16] |
| Hoesseinipour et al (2007) | 27 | 266 | 35 | 123 | 7.7% | 0.28 [0.16, 0.50] |
| Hailegebriel et al (2017) | 5 | 226 | 7 | 125 | 5.4% | 0.38 [0.12, 1.23] |
| Assefa et al (2009) | 7 | 214 | 13 | 164 | 6.2% | 0.39 [0.15, 1.01] |
| Roka et al (2012) | 11 | 260 | 5 | 50 | 5.6% | 0.40 [0.13, 1.20] |
| Escobado & Nunez (1999) | 0 | 67 | 2 | 136 | 1.7% | 0.40 [0.02, 8.42] |
| Abaver et al (2011) | 3 | 85 | 2 | 34 | 3.5% | 0.59 [0.09, 3.67] |
| Roka et al (2013) | 11 | 273 | 4 | 60 | 5.4% | 0.59 [0.18, 1.91] |
| Hailemariam et al (2004) | 2 | 78 | 1 | 26 | 2.4% | 0.66 [0.06, 7.57] |
| Moges et al (2006) | 6 | 28 | 100 | 376 | 6.3% | 0.75 [0.30, 1.91] |
| Mwambete et al (2010) | 10 | 66 | 15 | 80 | 6.5% | 0.77 [0.32, 1.86] |
| Tian et al (2012) | 11 | 302 | 13 | 303 | 6.7% | 0.84 [0.37, 1.91] |
| Babatunde et al (2010) | 7 | 90 | 7 | 90 | 5.7% | 1.00 [0.34, 2.98] |
| Feitosa et al (2001) | 16 | 365 | 198 | 5243 | 7.8% | 1.17 [0.69, 1.97] |
| Sanyaolu et al (2011) | 3 | 65 | 18 | 1015 | 5.1% | 2.68 [0.77, 9.34] |
| Akinbo et al (2011) | 74 | 2000 | 7 | 500 | 6.9% | 2.71 [1.24, 5.91] |
| Mariam et al (2008) | 6 | 109 | 1 | 51 | 2.8% | 2.91 [0.34, 24.85] |
| **Total (95% CI)** | | **6004** | | **8900** | **100.0%** | **0.59 [0.38, 0.92]** |
| Total events | 209 | | 450 | | | |

Heterogeneity: Tau² = 0.57; Chi² = 58.00, df = 19 (P < 0.00001); I² = 67%

Test for overall effect: Z = 2.34 (P = 0.02)

Odds Ratio M-H, Random, 95% CI

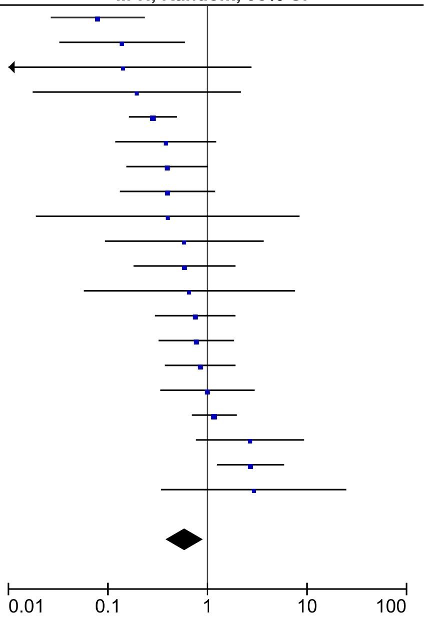


Supp. Figure 33

| Study or Subgroup | HIV positive SS positive Events | Total | HIV negative SS positive Events | Total | Weight | Odds Ratio M-H, Random, 95% CI |
|---|---|---|---|---|---|---|
| Udeh et al (2019) | 8 | 891 | 6 | 150 | 7.0% | 0.22 [0.07, 0.64] |
| Lebbad et al (2001) | 5 | 37 | 3 | 15 | 5.6% | 0.63 [0.13, 3.03] |
| Cerveja et al (2017) | 5 | 371 | 3 | 146 | 6.0% | 0.65 [0.15, 2.76] |
| Roka et al (2013) | 28 | 273 | 8 | 60 | 7.6% | 0.74 [0.32, 1.72] |
| Hailemariam et al (2004) | 4 | 78 | 1 | 26 | 4.1% | 1.35 [0.14, 12.66] |
| Marchiblatt & Cantos (2003) | 21 | 211 | 12 | 213 | 7.9% | 1.85 [0.89, 3.87] |
| Hoesseinipour et al (2007) | 2 | 266 | 0 | 123 | 2.8% | 2.33 [0.11, 48.99] |
| Moges et al (2006) | 3 | 28 | 15 | 376 | 6.3% | 2.89 [0.78, 10.64] |
| Abaver et al (2011) | 3 | 85 | 0 | 34 | 2.9% | 2.93 [0.15, 58.19] |
| Babatunde et al (2010) | 17 | 90 | 5 | 90 | 7.1% | 3.96 [1.39, 11.26] |
| Sanyaolu et al (2011) | 1 | 65 | 3 | 1015 | 4.0% | 5.27 [0.54, 51.39] |
| Mwambete et al (2010) | 2 | 66 | 0 | 80 | 2.8% | 6.24 [0.29, 132.29] |
| Hailegebriel et al (2017) | 39 | 226 | 4 | 125 | 7.0% | 6.31 [2.20, 18.10] |
| Getaneh et al (2010) | 23 | 192 | 4 | 192 | 7.0% | 6.40 [2.17, 18.87] |
| Mariam et al (2008) | 7 | 109 | 0 | 51 | 3.0% | 7.54 [0.42, 134.56] |
| Feitosa et al (2001) | 20 | 365 | 39 | 5243 | 8.3% | 7.74 [4.46, 13.41] |
| Roka et al (2012) | 23 | 260 | 0 | 50 | 3.1% | 9.99 [0.60, 167.25] |
| Akinbo et al (2011) | 23 | 2000 | 0 | 500 | 3.1% | 11.90 [0.72, 196.18] |
| Assefa et al (2009) | 27 | 214 | 1 | 164 | 4.6% | 23.53 [3.16, 175.11] |
| **Total (95% CI)** | | **5827** | | **8653** | **100.0%** | **2.71 [1.47, 5.00]** |
| Total events | 261 | | 104 | | | |

Heterogeneity: Tau² = 1.10; Chi² = 65.86, df = 18 (P < 0.00001); I² = 73%
Test for overall effect: Z = 3.19 (P = 0.001)

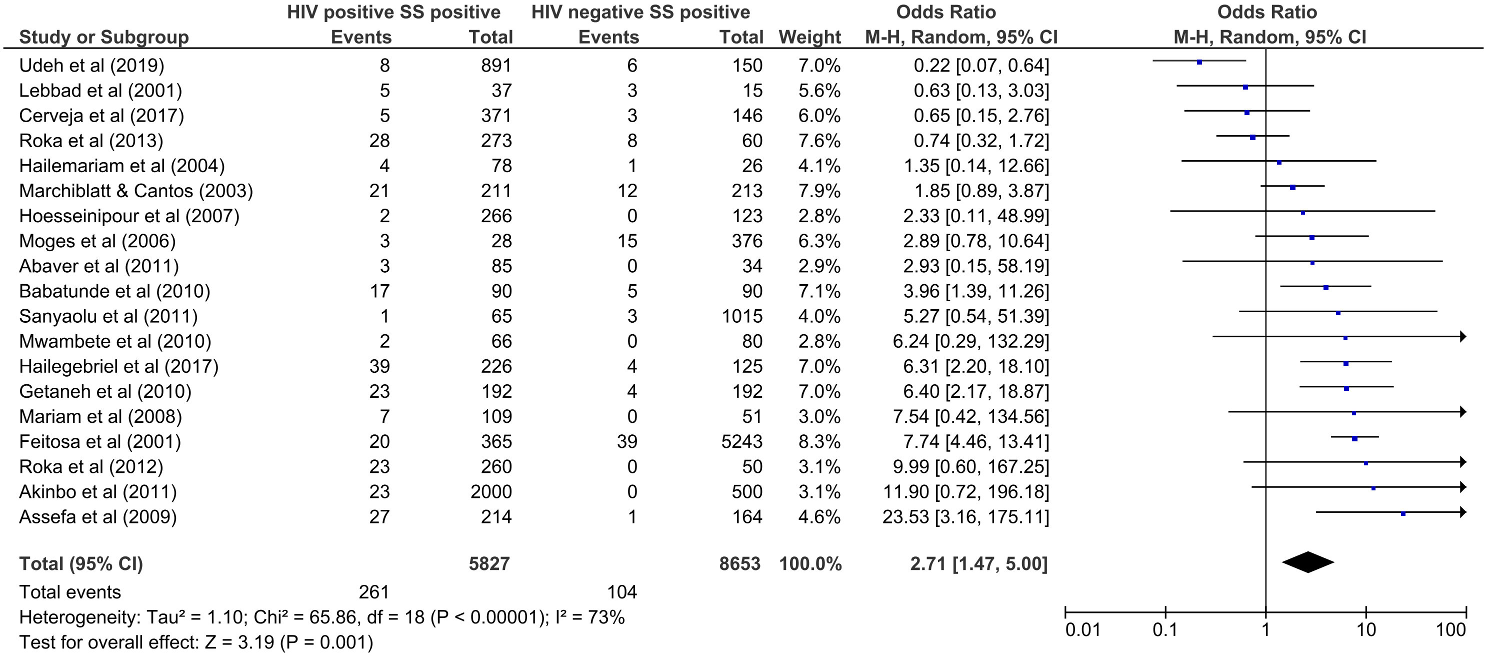


Supp. Figure 34
